# A Critical Overview of Current Theoretical Methods of Estimating the Energy of Intramolecular Interactions

**DOI:** 10.3390/molecules25235512

**Published:** 2020-11-25

**Authors:** Mirosław Jabłoński

**Affiliations:** Faculty of Chemistry, Nicolaus Copernicus University in Toruń, 87-100 Toruń, Poland; teojab@chem.umk.pl; Tel.: +48-056-611-4695

**Keywords:** intramolecular interaction, interaction energy, hydrogen bond

## Abstract

This article is probably the first such comprehensive review of theoretical methods for estimating the energy of intramolecular hydrogen bonds or other interactions that are frequently the subject of scientific research. Rather than on a plethora of numerical data, the main focus is on discussing the theoretical rationale of each method. Additionally, attention is paid to the fact that it is very often possible to use several variants of a particular method. Both of the methods themselves and their variants often give wide ranges of the obtained estimates. Attention is drawn to the fact that the applicability of a particular method may be significantly limited by various factors that disturb the reliability of the estimation, such as considerable structural changes or new important interactions in the reference system.

## 1. Introduction

Undoubtedly, intermolecular hydrogen bonds [1,2,3,4,5,6,7,8,9,10,11,12,13,14,15,16] occupy the main place among various intermolecular interactions. This is largely due to their intermediate strength, between weaker van der Waals interactions [7,11] and much stronger chemical bonds [1,17]. It is this intermediate strength of intermolecular hydrogen bonds that allows for them to act as a glue that binds various molecules into dimers or larger molecular aggregates. On the other hand, their relative weakness allows for the full dynamics of the bonding motif; the hydrogen bond can be broken relatively easily and a new one can be formed in its place.

It is already visible at this point that the knowledge of the strength of intermolecular hydrogen bonding is a very important element in the full description of the characteristics of this bond. Such knowledge would allow, for example, to classify them according to the strength found and study the impact of various internal and external factors on it. Because of the fact that the total energy of a molecule is a fundamental quantity available to quantum mechanics [18], the appropriate balance of total energies can be successfully used to write a strict definition of the energy of interaction (i.e., the interaction energy) between A and B systems in AB dimer:

(1) $$E_{\mathrm{int}}=E\left(\mathrm{AB}\right)-\left[E\left(A\right)+E\left(B\right)\right]$$

Therefore, it is clear that the reference system for the bound AB dimer is that of the isolated monomers A and B. Another thing that I will leave behind is that these monomers may have their own, i.e., fully optimized geometries or geometries taken from the dimer. Anyway, the energy that is described by Equation (1) is strictly defined. Not quite rightly, $E_{\mathrm{int}}$ obtained by Equation (1) is commonly taken as the interaction energy associated with the closest contact between A and B, e.g., an intermolecular hydrogen bond. Therefore, this equation has also become the main source of information regarding the strength of intermolecular hydrogen bonds in the so-called supermolecular method.

It is quite natural that one would like to have such an important quantity also in the case of intramolecular interactions, including intramolecular hydrogen bonds. However, there is a fundamental problem here. Namely, unlike in its intermolecular counterpart, breaking the intramolecular interaction is impossible without disturbing the structure of the molecule. Because of this fact, not only it is impossible to find a strict definition of the intramolecular interaction energy, but what is more, this energy is not even strictly definable (see, however, the further discussion on the QTAIM-based methods).

Nevertheless, one can try to introduce a method that results in a number that is treated (in this method) as the energy of a given intramolecular interaction. It is obvious that, in the general case, the energies obtained will differ (perhaps even significantly) among the adopted methods. For this reason, an important aspect of the proposed method is the evaluation of the reliability of the energy obtained. It would rather be a worthless result to obtain for intramolecular hydrogen bond of e.g., the OH⋯O type an energy of the order of, say −50 kcal/mol, if the intermolecular equivalent in the case of a similar configuration of O and H atoms gives energy from about −4 to about −8 kcal/mol. One of the possible ways of assessing the reliability of the obtained energy value is thus comparing it to the appropriate intermolecular interaction, in which not only the type of X and Y atoms (from the XH⋯Y contact), but also their spatial configuration (e.g., the key distance H⋯Y) is largely preserved. Another sensible possibility is to check the fulfillment of various correlations between the found energy values and other parameters describing the strength of the H⋯Y bond. One should also compare the obtained estimates for structurally closely related molecules.

This article reviews the current theoretical methods introducing the concept of the XH⋯Y intramolecular hydrogen bond interaction energy (or more generally the intramolecular X⋯Y interaction) and allowing for the computational generation of these energies. The main emphasis will be on the problems associated with these methods, which may naturally lead to their different, more or less reliable, variants. On the other hand, due to the multitude of numerical data concerning the interaction energy values that were determined with these methods, this issue will necessarily be of minor importance. Rather, I will limit myself to a few examples that illustrate how a given method works.

## 2. Theoretical Methods of Estimating the Energy of Intramolecular Interactions

### 2.1. Conformational Methods

As noted in the Introduction, it is impossible (see, however, the further discussion on the QTAIM-based methods) to precisely define the energy of the intramolecular hydrogen bond XH⋯Y (or more generally of the intramolecular interaction X⋯Y), because it is impossible to create a reference system in which there would be no such interaction, but in which the configuration of all atoms would be preserved. In such a reference system, the interaction of interest would be simply “switched off”. Crucially, this approach is eqivalent to the following partition of the total energy of the system (the so-called closed or chelate form) containing the interaction of interest (e.g., a hydrogen bond)

(2) $$E\left(\mathrm{closed}\right)=E^{f}\left(\mathrm{closed}\right)+E_{\mathrm{HB}}$$

in which E(closed), $E^{f}\left(\mathrm{closed}\right)$, and $E_{\mathrm{HB}}$ correspond successively to the total energy of the closed form, the total energy of a fictitious closed form with the interaction switched off, and the hydrogen bond interaction energy. Of course, such an exclusion is impossible, but, nevertheless, one may be tempted to find another system being very similar to the fictitious closed one. Due to the fact that total energy depends on the number and type of particles making up a given molecule, the phrase “another but very similar system” should be understood as a different conformer of the closed form of a molecule. This leads to so-called conformational methods, i.e., methods which use total energies of at least two conformers of a molecule having the intramolecular interaction.

#### 2.1.1. The Open-Closed Method (OCM)

The simplest and the most frequently used method of estimating the energy of intramolecular interactions, including intramolecular hydrogen bonds, is the so-called open-closed method (OCM) [5,19]. Apart from the molecule that contains a given interaction (i.e., the closed or chelate form), OCM requires the use of one more reference form (the so-called open), in which this interaction is absent [20,21,22,23,24,25,26,27,28,29,30,31,32,33,34,35,36,37,38,39,40,41,42,43,44,45]. It is then assumed that

(3) $$E^{f}\left(\mathrm{closed}\right)\approx E\left(\mathrm{open}\right)$$

which means that the total energy of another conformer, i.e., the open form, can be used instead of the impossible to obtain total energy of the fictitious closed system. Substituting expression (3) to (2) leads to a simple expression for the intramolecular hydrogen bond energy in OCM:

(4) $$E_{\mathrm{HB}}^{\mathrm{OCM}}=E\left(\mathrm{closed}\right)-E\left(\mathrm{open}\right)<0$$

It should be emphasized that this article adopts the convention according to which a negative value of the obtained interaction energy means local stabilization that results from H⋯Y, while on the contrary, a positive value means local destabilization. Thus, of course, as being stabilizing interactions, hydrogen bonds should be characterized by negative values.

**Figure 1:**
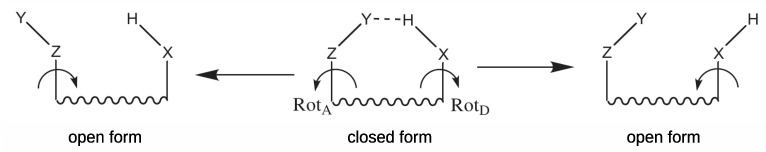
Scheme showing two open forms obtained by rotation of either the hydrogen-acceptor (lhs) or the hydrogen-donor (rhs) group.

Equation (3) requires that the open form does not differ much from the (fictitious) closed form. Therefore, the open form is most often obtained by rotating the donor or acceptor group by 180${}^{\circ }$, as shown in Figure 1.

It is understood that, in general, these reference open forms give different values of $E_{\mathrm{HB}}^{\mathrm{OCM}}$ [33]. In principle, one can also try to use a different open form. However, I will come back to this issue further. Although the expression (3) suggests that the open form should be fully optimized, i.e., it should correspond to a local minimum on the potential energy hypesurface, another possibility is to use an open form having the geometry (more precisely, geometrical parameters) of the closed form [5,19,33,38,39,42,44,45]

(5) $$E^{f}\left(\mathrm{closed}\right)\approx E^{\mathrm{closed}}\left(\mathrm{open}\right)$$

**Figure 2:**
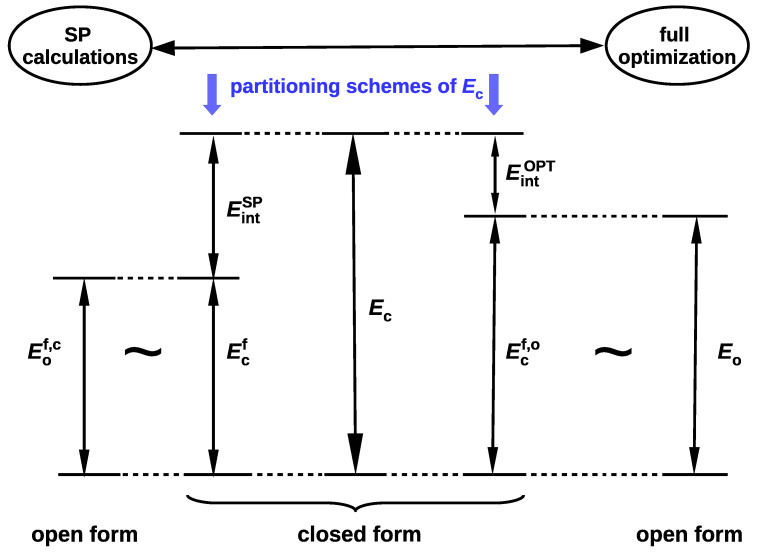
Scheme showing two variants of the closed form total energy partition ($E_{c}$) to the interaction energy ($E_{\mathrm{int}}$) and the total energy of the fictitious closed form ($E_{c}^{f}$) obtained after ‘excluding’ this interaction.

Of course, this leads to a different energy value

(6) $$E_{\mathrm{HB}}^{\mathrm{OCM}}=E\left(\mathrm{closed}\right)-E^{\mathrm{closed}}\left(\mathrm{open}\right)$$

since $E^{\mathrm{closed}}\left(\mathrm{open}\right)\neq E\left(\mathrm{open}\right)$. In fact, Schuster advocated this option, suggesting that the reference open form should have "the least changes in molecular geometry besides a cleavage of the H-bond” and proclaiming that it "need not be a local minimum of the energy surface” [5]. Moreover, in his opinion, the full optimization of the open form geometry is even inadvisable, because this approach mixes the energy of isomerization (resulting from the change of the conformer) into the determined energy value [5]. In fact, both of these approaches introduce different definitions of the intramolecular interaction energy (cf. Equations (4) and (6)). This situation is somewhat similar to the one that occurs when determining the interaction energy from Equation (1). Namely, the use of the monomers A and B with their geometries taken from the AB dimer defines the interaction energy, while their full optimization leads to the binding energy. The latter quantity also takes into account the correction for geometry change that takes place during the transition from the isolated form to the bound form in the dimer. Because of the fact that, in OCM, the fictitious closed form is replaced by the open form obtained by some conformational change, Schuster stressed that any splitting of the energetical difference between both forms is artificial [5]. However, it seems that this opinion may be slightly weakened by some corrective approaches [39,44]. It is valuable to present both variants of the partition of the total energy of the closed form in one scheme, as shown in Figure 2, where more concise notations are used for the respective energies.

It is worth noting that $|E_{o}\left|>\right|E_{o}^{f,c}|$, which, in principle, should lead to the relationship $|E_{\mathrm{int}}^{\mathrm{OPT}}\left|<\right|E_{\mathrm{int}}^{\mathrm{SP}}|$. It seems that at present the variant based on full geometry optimization of the open form (leading to $E_{o}$ and then $E_{\mathrm{int}}^{\mathrm{OPT}}$) is much more popular [37] than the variant based on single point calculations (leading to $E_{o}^{f,c}$ and then $E_{\mathrm{int}}^{\mathrm{SP}}$). In this variant, the isomerization energy mentioned by Schuster [5] is ‘absorbed’ into the interaction energy. In other words, this variant assumes that the changes in geometrical parameters that take place during the open form → closed form transition are related to the continuous process of creating the interaction (e.g., an intramolecular hydrogen bond) in the closed form [42,45].

**Figure 3:**
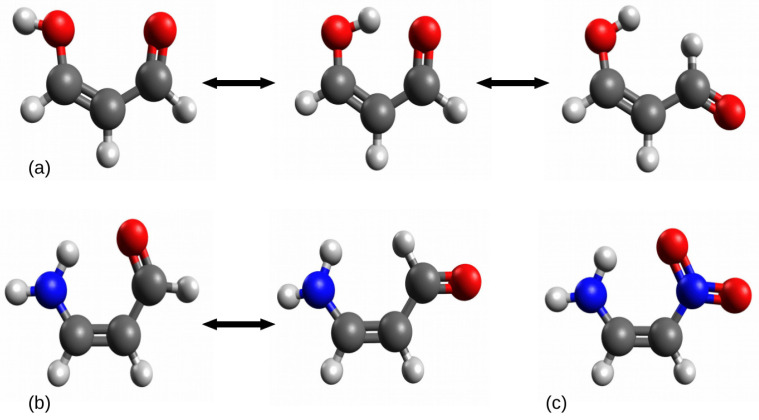
Examples of problematic cases in the open-closed method: (**a**) malondialdehyde, (**b**) 3-aminoacrolein, (**c**) 1-amino-2-nitroethylene.

Although OCM seems to be the most frequently [20,21,22,23,24,25,26,27,28,29,30,31,32,33,34,35,36,37,38,39,40,41,42,43,44,45] used theoretical method of estimating the energy of an intramolecular interaction, it is not free from further problems. The rotation of the proton-donor or the proton-acceptor group quite often leads to a new, significant interaction (either repulsive or attractive) in an open form [24,27,28,29,31,33,39,40,41,42,43,44,45,46]. Unfortunately, this possibility is quite often ignored. Moreover, sometimes, one or even both of the open forms cannot be used due to symmetry of these groups. Some simpe examples representing both cases are shown in Figure 3. Of course, similar examples can be easily invented endlessly.

In the case of (a) relating to the intramolecular hydrogen bond O-H⋯O in malondialdehyde, the rotation of the proton-donor group -OH leads to a new rather significant interaction O⋯O, while the rotation of the proton-acceptor group -CHO leads to also rather significant new interaction H⋯H. In the case of (b) (3-aminoacrolein), due to the symmetry of the amino group, its rotation leads to practically the same system, while the rotation of the aldehyde group leads to a new significant H⋯H interaction, similar to the case of (a). The closed form of 1-amino-2-nitroethylene does not have any such simple open forms due to the symmetry of both groups, i.e., -NH_2_ and -NO_2_.

Another, but important, question is whether these new interactions can be completely ignored [24,27,28,29,33,39,40,41,42,43,44,45,46]. For example, in the case of malondialdehyde, geometry optimizations (B3LYP/aug-cc-pVTZ) of the open form shown on the left-hand side of Figure 3 gives 2.89 Å for the O⋯O distance and 2.02 Å for H⋯H in the open form shown in the right-hand side of this figure. In the case of the open form of 3-aminoacrolein, the distance H⋯H is 2.18 Å. Therefore, it would seem that these distances are too large for the interaction energy to be uncertain. However, on the other hand, the comparison of the CCC angle values in both forms (119.6${}^{\circ }$ vs. 126.8${}^{\circ }$ and 125.2${}^{\circ }$ in malondialdehyde and 122.0${}^{\circ }$ vs. 125.1${}^{\circ }$ in 3-aminoacrolein) shows that the closed form → open form transition leads to an opening of the molecular skeleton, which may suggest significant repulsive actions of both these interactions. It seems that the O⋯O contact, in particular, cannot be completely ignored here. It is worth mentioning that both forms, i.e., closed and open, may differ in some structural aspects, e.g., the amino group in 3-aminoacrolein (b) is flat in the closed form, whereas slightly pyramidal in the optimized open form. In this case, one would have to decide whether the pyramidalization energy of the amino group should be shelled out or included in the hydrogen bond energy value [47].

**Figure 4:**
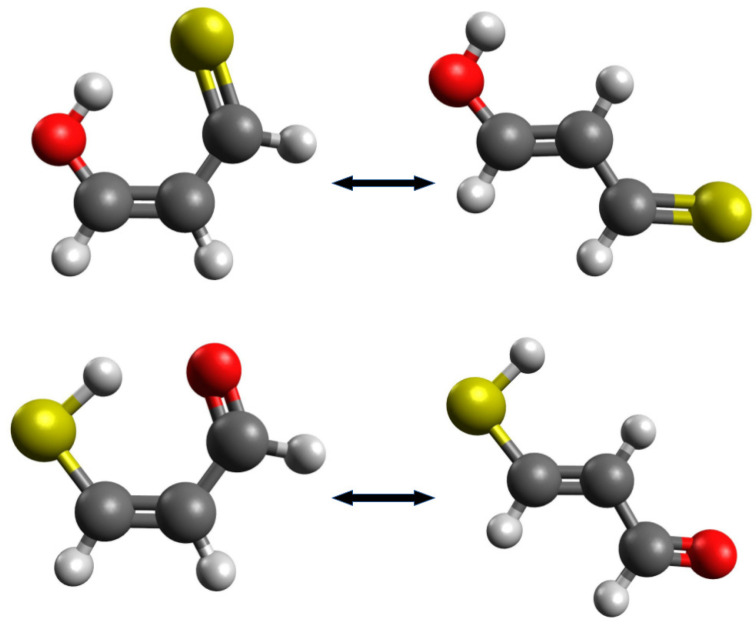
Closed and the most extended enol and enethiol forms of thiomalondialdehyde.

In such and similar cases, it may be tempting to find other reasonable open forms, obtained after the rotation of one of the groups around the CC double bond. On the one hand, such new interactions will be avoided, but on the other hand, the configuration of the carbon skeleton of the molecule will be changed. For example, Buemi et al. [33] rebuked the use of the most extended enol and enethiol tautomers of thiomalondialdehyde [48,49] as reference structures [24,50], because, in their opinion, the *trans* configuration of double bonds seems to be too different that the *cis* arrangement in the closed form (Figure 4).

It is also worth adding that the most extended conformers are very often the global minima of a given molecule. On the other hand, open systems with a changed configuration of backbone atoms can be more reasonable in many cases. In fact, the selection of the most reasonable reference system is an individual matter for the closed form of the molecule under consideration. Therefore, this issue should be carefully analyzed before starting the appropriate calculations while using OCM.

**Figure 5:**
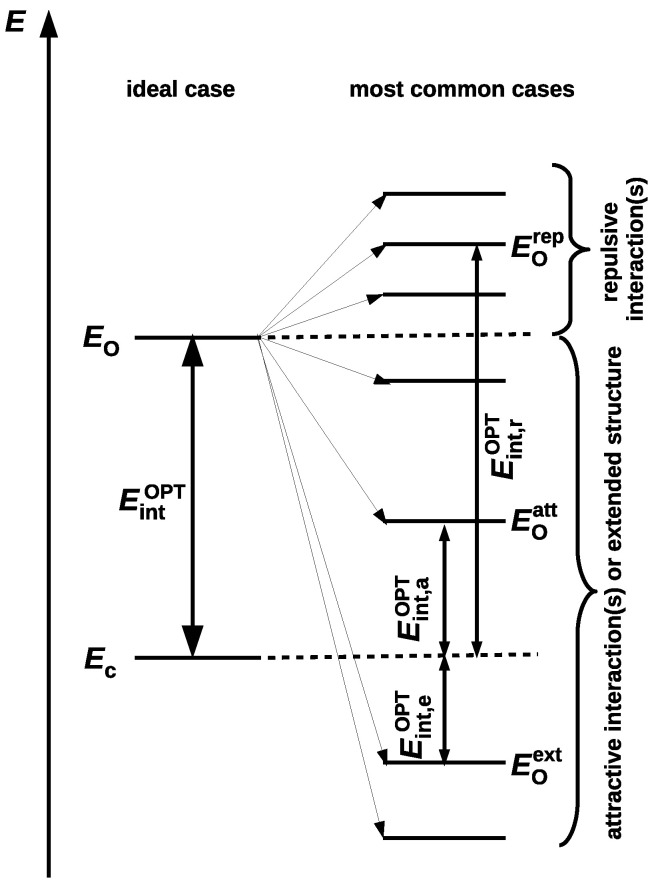
Scheme showing the presence of a new either repulsive or attractive interaction as a cause of either overestimating or underestimating the determined value of the intramolecular interaction energy.

The fundamental issue for OCM is that the presence of a new significant interaction in the reference open form leads to either an overestimation or underestimation of the determined value of the interaction energy in the closed form [42,45]. Both of the situations are shown in Figure 5.

The presence of a new significantly repulsive interaction in the reference open form leads to a less negative total energy of this form ($E_{o}^{\mathrm{rep}}$), and thus to an overestimation of the determined value of the interaction energy ($E_{\mathrm{int},r}^{\mathrm{OPT}}>E_{\mathrm{int}}^{\mathrm{OPT}}$). Conversely, the presence of a significant attractive (stabilizing) interaction, e.g., a new hydrogen bond, results in underestimating the determined energy value ($E_{\mathrm{int},a}^{\mathrm{OPT}}<E_{\mathrm{int}}^{\mathrm{OPT}}$). Moreover, because the most extended forms are often the most stable (as already mentioned), $E_{o}^{\mathrm{ext}}<E_{c}$, their frivolous use can underestimate the value of the interaction energy so much that this value can even change the sign ($E_{\mathrm{int},e}^{\mathrm{OPT}}$) [42]. As open forms with presence of new locally repulsive interactions X⋯Y (e.g., O⋯O, O⋯S, S⋯S, etc.) and, in particular, H⋯H are often treated favorably, the resulting energies may often be overestimated. This, in turn, may lead to overinterpretations of the considerable strength of some intramolecular hydrogen bonds [39].

Given the fact that the full geometry optimization of the open form can lead to a new significant interaction (repulsive or attractive) or to a significant change in structure as compared to the closed form, a solution may be to perform a partial (i.e., constrained) geometry optimization [42]. In many cases, it is enough to ‘freeze’ one or two dihedral angles that define the spatial orientation of the proton-donor or proton-acceptor group, the optimization of which would lead to the previously mentioned undesirable effects. However, sometimes, it is also necessary to freeze other geometric parameters [42]. The approach that is based on partial geometry optimization of the open form is, in fact, another variant of OCM, leading to the interaction energy value between these described by Formulas (4) and (6).

**Figure 6:**
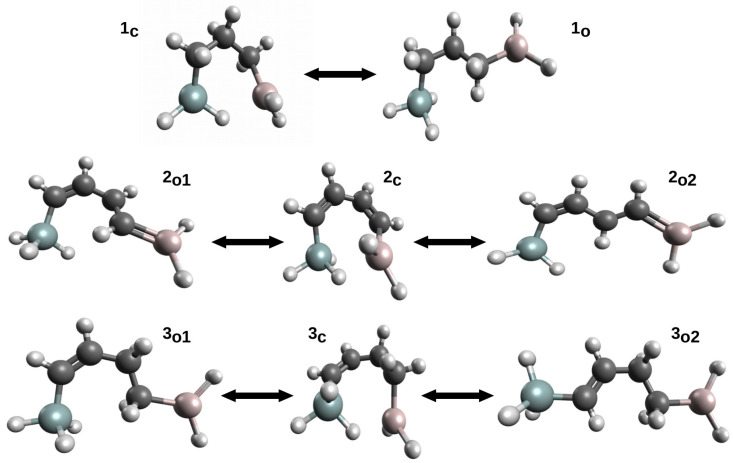
Closed and some open forms of (**1**) H_3_Si-CH_2_-CH_2_-CH_2_-AlH_2_, (**2**) H_3_Si-CH=CH-CH=CH-AlH_2_ and (**3**) H_3_Si-CH=CH-CH_2_-CH_2_-AlH_2_.

This variant was first proposed [42] to estimate the energy of Si-H⋯Al intramolecular charge-inverted hydrogen bonds [51,52] in ten model systems. The energy values of Si-H⋯Al in these systems were determined while using seven variants of OCM. In addition to either the full optimization (OPT) or complete freeze (SP) of the open form geometry, five variants of the constrained optimizations of the open form geometry were also used: (P1) only bonds optimized, (P2) only bonds and plane angles optimized, (P3) all geometric parameters optimized but dihedral angles governing the positions of the Si atom and the -AlH_2_ group in relation to the carbon skeleton of the reference form, (P4) all geometric parameters optimized but dihedral angles governing the positions of the Si atom and both hydrogen atoms from the -AlH_2_ group, and (P5) all geometric parameters optimized, but dihedral angles governing the positions of both hydrogen atoms from -AlH_2_. Of course, the values of the non-optimized geometric parameters in the variants SP and P1–P5 were taken from the closed form. Therefore, it can be seen that the P1–P2 variants in a controlled manner increase the number of optimized parameters (degrees of freedom), which increases the flexibility of the approach. Because the obtained results [42] very well reflect the mentioned problems related to the use of OCM, these results are shown for three molecules (Figure 6) in Table 1.

First of all, it can be seen that the determined values of the interaction energy vary widely, depending on the variant of the open-closed method used in the calculations. In the case of molecule **1**, it is from −7 to −1 kcal/mol and, in the case of **3**, from −10.6 to about −0.8 kcal/mol. The values decrease (i.e., become less negative) with an increased degree of flexibility regarding the geometric parameters optimized in a given variant. It can be seen that especially even a partial optimization of dihedral angles has a large influence on the determined interaction energy values. Moreover, the rotation of the -SiH_3_ group in general gives significantly different values from that when the −AlH_2_ group is rotated. This is especially visible for the least flexible variant SP, while on going from P1 to P5 these differences become smaller and smaller. It is instructive to analyze the results from the last column of Table 1, i.e., regarding the variant with full geometry optimization of the proposed open form. While in case of **1** one reasonable value was found (−1.08 kcal/mol), in the case of **6** two significantly different values were obtained (−4.97 and −0.75 kcal/mol). The latter results from the fact that two open reference forms (see $3_{o1}$ and $3_{o2}$ in Figure 6) with quite different characteristics were obtained. Despite the fact that both forms have identical carbon frame configuration (*cis*), the $3_{o2}$ form has two new H${}^{\delta +}\cdots$H${}^{\delta +}$ interactions. On the other hand, the $3_{o1}$ form has two pairs of probably less important H${}^{\delta -}\cdots$H${}^{\delta +}$ interactions. Case **2**, on the other hand, is an important example illustratating the significant impact of the presence of a completely new type of interaction in an open form on the quality of the estimation of the interaction energy is a closed form. Namely, in both open forms ($2_{o1}$ and $2_{o2}$), the -AlH${}_{2}$ group (Al has an empty *p* orbital) takes a coplanar position to the CH=CH fragment with a formal C=C double bond. This arrangement allows for the $p_{\pi }\to$Al coupling (highlighted in Figure 6 by drawing a C=Al double bond), which significantly lowers total energies of these forms. Consequently, the estimates of the interaction energy of Si-H⋯Al in $2_{c}$ are highly unreliable.

**Figure 7:**
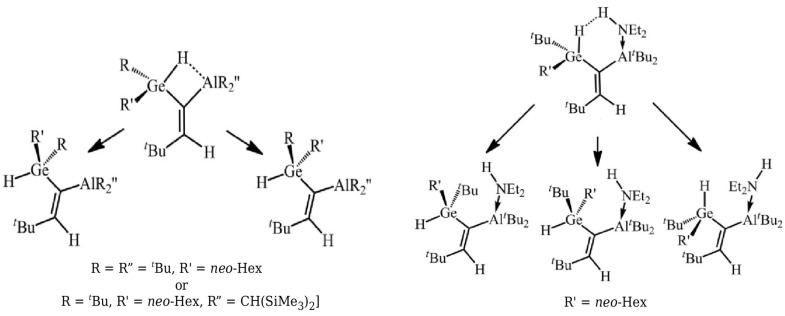
Closed and open forms investigated in ref. [46].

The variant of OCM with partial geometry optimization of the open form was then used [45] to estimate energies of Si-H⋯B contacts in some 1-silacyclopent-2-enes and 1- silacyclohex-2-enes and helped to successfully support the earlier Wrackmeyer’s suggestion based on NMR spectroscopic data [53] that this contact is considerably stronger in the latter system than in the former one. Additionally, the energies of Ge-H⋯Al and Ge-H⋯H-N interactions in some alkenylhydrogermanes were estimated [46] in a similar way (see Figure 7).

The variant of OCM with partial geometry optimization of the open form should rather be treated as a certain, but probably not the only, possible solution when the full geometry optimization of this form gives (for the reasons discussed earlier) highly unreliable estimates of the interaction energy [42,45].

The results that are presented here are enough to show that OCM in which only one reference system is utilized must be used with great caution so as not to write with reserve. It should be so especially when its—nevertheless the most popular—variant with the full geometry optimization of the reference open form is used. Not as rare as it may seem at first, the occurrence of new interactions (whatever attractive or repulsive) or significant structural changes (e.g., changing the skeleton of a molecule) can lead to highly unreliable estimates of the energy value of the intramolecular interaction of interest in a closed form. Indeed, Rozas et al. [32] went so far as to say that the energy value obtained from OCM should scarcely be taken as the value of the energy of the interaction in a closed form. Simply, it should rather be treated as the energetical difference between the respective forms of a molecule. On the other hand, this criticism seems a bit exaggerated. If the open form is very similar to the closed form both in terms of structure and the interactions occurring in these forms, then it seems that OCM is a worthy method of choice. The substantial similarity that is referred to herein can be provided by the presence of some rigid part of the molecule to which both the donor and acceptor groups are attached. This is the case, for example, with a benzene ring, leading to the variant of OCM, described as the *ortho*-*para* method [38,54,55], which is described in more detail in the next subsection.

#### 2.1.2. Ortho-Para Method (opM)

**Figure 8:**
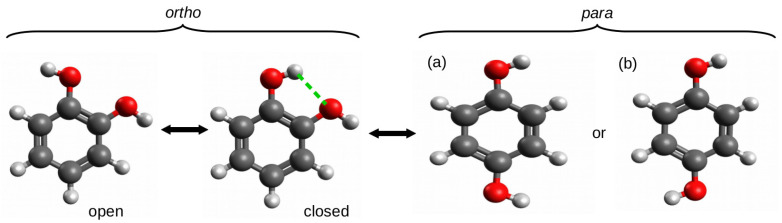
Various forms of catechol (the subfigures (**a**) and (**b**) represent different forms of *para*-catechol).

The *ortho*–*para* method (opM) was most likely used for the first time by Estácio et al. [38] for estimating the energy of intramolecular hydrogen bonds in four 1,2-disubstituted benzene derivatives: 1,2-dihydroxybenzene (catechol), 1,2-benzenedithiol, benzene-1,2-diamine, and 2-methoxyphenol (guaiacol). To describe opM, it is enough to refer to the O-H⋯O hydrogen bond in 1,2-dihydroxybenzene, i.e., catechol (Figure 8).

The use of the open form that was simply obtained by rotating the hydroxyl group around the C-O bond resulted in hydrogen bond energy estimates of −3.7 or −4.0 kcal/mol at the MPW1PW91/aug-cc-pVDZ and CBS-QMPW1 levels of theory, respectively. These values were considered to be unreliable and significantly overestimated as a result of the presence of new repulsive interactions between oxygen atoms as well as the O-H dipole–dipole interactions [38]. As a consequence, it was concluded that the energetic difference between the open and closed forms cannot be regarded as the energy of the O-H⋯O hydrogen bond in the latter form. However, in this and similar cases, the *para* form is a very reliable reference form. The comparison of total energy of this form with the total energy of the closed form of the *ortho* configuration gives opM, which can be seen as a variant of OCM. Based on this approach, the respective hydrogen bond energies were −2.1 and −2.3 kcal/mol [38].

It is worth emphasizing here that the high reliability of the estimate obtained by means of opM results from the high stiffness of the main part of the molecule, i.e., the benzene ring and, hence, the significant transferability of the related geometric parameter values. In other words, the stiffness of the molecular framework and its high preservation when going to the *para*-substituted reference system allowed for avoiding the typical problems that are faced by the standard version of OCM which were mentioned earlier. On the other hand, it should be noted that this method assumes that the substituent electronic effects in the *ortho* and *para* forms are similar. However, this is in line with the general knowledge on substituent effects [56,57,58,59]. Nevertheless, another question, which is completely not addressed by Estácio et al., is which form of the *para* conformer (see (a) and (b) in Figure 8) to use. While this rather purely theoretical issue seems to be insignificant for catechol due to the negligible difference in total energies between the two forms (e.g., 0.1 kcal/mol at the B3LYP/aug-cc-pVTZ level of theory), the difference may become slightly larger for other substituents or molecular frameworks.

It should be mentioned that Estácio et al. described the O-H⋯O hydrogen bond in the closed form of catechol by means of a simple model that is based on the description of interacting dipoles of the O-H bonds. This model resulted in the following formula

(7) $$E_{\mathrm{HB}}=-\left[E_{\mathrm{LJ}}^{O\cdots O}+E_{\mathrm{dd}}\right]$$

where $E_{\mathrm{LJ}}^{O\cdots O}$ is the Lennard–Jones interaction energy for the relevant pair of oxygen atoms and $E_{\mathrm{dd}}$ is the dipole-dipole interaction energy for the closed form [38]. The energy value that was determined using this formula was −2.0 kcal/mol (MPW1PW91/aug-cc-pVDZ) and it was very closed to the one determined while using opM (−2.1 kcal/mol). Estácio et al. considered this result to be significant, because it shows that opM correctly describes both interactions, i.e., the O⋯O repulsion and the interaction between the dipoles of both O-H bonds in the closed form of catechol.

#### 2.1.3. Related Rotamers Method (RRM)

**Figure 9:**
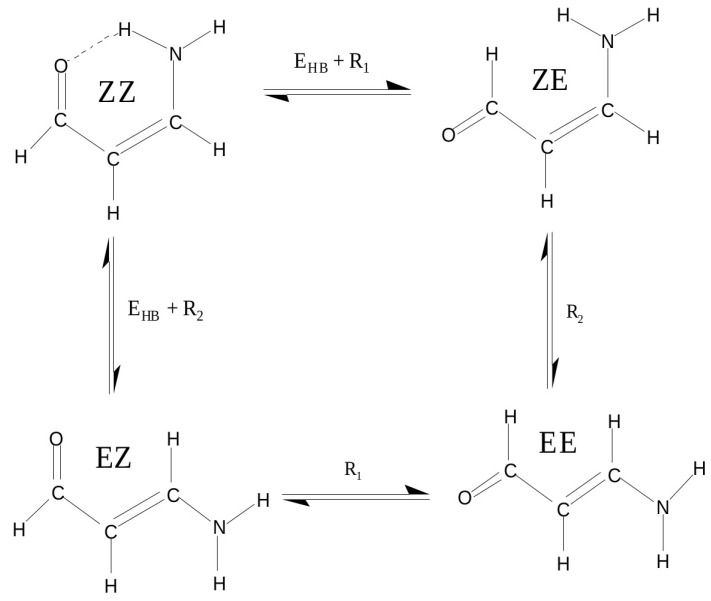
Four conformers of 3-aminoacrolein and energetic relationships between them.

As we have seen, the choice of a reasonable open form in OCM is often problematic and even sometimes impossible. This is due to the requirement that this form should be as close to the closed form as possible. This means that the conformer change should not lead to significant changes in the values of geometric parameters. In order to overcome any inaccuracies, another approach is to use more than just two conformers of a given molecule [47,60,61,62,63]. This idea will be shown on the example of 3-aminopropenal (3-aminoacrolein), which has four conformers. The N-H⋯O hydrogen bond energy in the ZZ conformer of 3-aminoacrolein was quite often estimated [47,61,63,64,65], but the methods used did not take into account changes in the values of geometric parameters when switching from the bound system (ZZ-3-aminoacrolein) to reference forms (in particular, to ZE-3-aminoacrolein) [64,65]. The specific system of conjugated double bonds and, hence, the presence of four conformers (see Figure 9), allowed for proposing a method that was derived from the analysis of the mutual energy relations between the four conformers of 3-aminoacrolein (Figure 9) [47].

This method takes use of approximations

(8) $$E_{\mathrm{HB}}+R_{1}=E^{\mathrm{ZZ}}-E^{\mathrm{ZE}},\;\;\;\;\;\;R_{1}\approx E^{\mathrm{EZ}}-E^{\mathrm{EE}}$$

(9) $$E_{\mathrm{HB}}+R_{2}=E^{\mathrm{ZZ}}-E^{\mathrm{EZ}},\;\;\;\;\;\;R_{2}\approx E^{\mathrm{ZE}}-E^{\mathrm{EE}}$$

that lead to the following formula for the hydrogen bond energy in the ZZ form of 3-aminoacrolein

(10) $$E_{\mathrm{HB}}^{\mathrm{RRM}}=\left(E^{\mathrm{ZZ}}-E^{\mathrm{ZE}}\right)+\left(E^{\mathrm{EE}}-E^{\mathrm{EZ}}\right)$$

Calculations that are based on MP2(Full)/6-31G** and MP2(FC)/6-311+G** level of theory gave values of −8.2 and −7.5 kcal/mol, respectively [47]. Later, B3LYP/6-311++G** (however, most likely Nowroozi et al. [61] used a smaller 6-31G** basis set, as evidenced by the number of 100 basis functions mentioned by them and the obtained value of −8.4 kcal/mol, which is close enough to the value of −8.2 kcal/mol obtained [47] at the MP2(Full)/6-31G** level of theory) computation by Nowroozi et al. [61] gave value of −8.4 kcal/mol.

The term in the first bracket of Equation (10) is equivalent to the energy that is obtained from the most commonly used variant of OCM in which the open reference form is obtained by the rotation of the proton-acceptor group. Hence, the relationship between RRM and OCM can be expressed by the following relationship between the total energies of the conformers EE and EZ [44]:

(11) $$E_{\mathrm{HB}}^{\mathrm{RRM}}-E_{\mathrm{HB}}^{\mathrm{OCM}}=E^{\mathrm{EE}}-E^{\mathrm{EZ}}$$

Because, in most cases, the extended EE conformer is more stable than the EZ conformer, the difference defined by the above equation is negative. For this reason, as compared to OCM, RRM gives greater stabilizations of interactions. It may even happen that interactions that are weakly destabilizing based on OCM are weakly stabilizing if RRM is considered instead, and this result is only due to “different zeros” in both of these methods [44].

It should be mentioned that RRM [47,60] has been readily adopted by Nowroozi et al. [61,62,63], who called it the Related Rotamers Method (RRM) and in this review it functions under that name. However, even a little earlier, practically the same method was used by Lipkowski et al. [60] to estimate the energy of O-H⋯N intramolecular hydrogen bonds in some chloro-derivatives of 2-(N-dimethylaminomethyl)-phenols, but they used the term “thermodynamic cycle”. Therefore, as one can see, not only are different methods used, but even the same method can function under different names [47,60,61,62,63].

#### 2.1.4. Geometry-Corrected Method (GCM)

All of the methods for estimating the energy of intramolecular interactions (e.g., hydrogen bonds) discussed so far do not take into account changes in the values of geometric parameters upon considering an open reference form of a molecule. However, the presence of a conjugated system of double bonds, which is characteristic for 3-aminoacrolein and, thus, the existence of its four conformers (Figure 9) allowed for proposing a method to estimate the energy of the N-H⋯O intramolecular hydrogen bond in the ZZ conformer with simultaneous partial consideration of geometric factors [39]. This method initially functioned under the name “Scheme A” [39,40,41,43], but later its meaningless name was changed to the Geometry-Corrected Method (GCM) [44].

**Figure 10:**
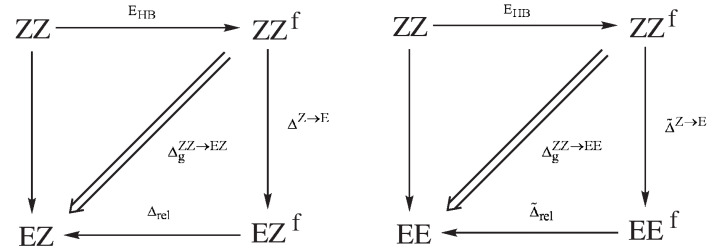
Energy dependencies between the respective forms of 3-aminoacrolein used in deriving the formula for the energy of the intramolecular N-H⋯O hydrogen bond in the ZZ form, according to Geometry-Corrected Method (GCM).

Very helpful in understaning the idea of GCM and how to derive it are the diagrams presented in Figure 10, which show the energy relationships between the respective forms of 3-aminoacrolein.

As in Equation (2), in the first step, it is assumed that the hydrogen bond in the ZZ form of 3-aminoacrolein can be simply ’turned off’ without any changes in the electron density distribution of the system, therefore also without any changes in the geometrical parameters of this form. By introducing an approximation of the energy additivity, we obtain:

(12) $$E_{\mathrm{HB}}=E^{\mathrm{ZZ}}-E^{\mathrm{ZZ},f}<0$$

where $E^{\mathrm{ZZ},f}$ is simply the total energy of the fictitious form of ZZ with the hydrogen bond just ‘turned off’. The rotation of the aldehyde group around the C=C double bond, i.e., the transition ZZ→EZ leads not only to breaking the hydrogen bond, but also to some changes in the geometrical parameters. If the energy associated with these changes in geometric parameters is $\Delta_{g}^{\mathrm{ZZ}\to \mathrm{EZ}}$, then

(13) $$E^{\mathrm{EZ}}\approx E^{\mathrm{ZZ}}+E_{\mathrm{HB}}+\Delta_{g}^{\mathrm{ZZ}\to \mathrm{EZ}}=E^{\mathrm{ZZ},f}+\Delta_{g}^{\mathrm{ZZ}\to \mathrm{EZ}}$$

and quite similarly for the ZZ→EE transition

(14) $$E^{\mathrm{EE}}\approx E^{\mathrm{ZZ}}+E_{\mathrm{HB}}+\Delta_{g}^{\mathrm{ZZ}\to \mathrm{EE}}=E^{\mathrm{ZZ},f}+\Delta_{g}^{\mathrm{ZZ}\to \mathrm{EE}}$$

Dividing the sum of Equations (13) and (14) by two, one obtains an expression that can be interpreted as the averaged energy that is related to the configuration change Z→E:

(15) $$\Delta_{g}^{\mathrm{av}}=\frac{1}{2}\left(\Delta_{g}^{\mathrm{ZZ}\to \mathrm{EZ}}+\Delta_{g}^{\mathrm{ZZ}\to \mathrm{EE}}\right)=\frac{1}{2}\left(E^{\mathrm{EZ}}+E^{\mathrm{EE}}\right)-E^{\mathrm{ZZ},f}$$

Combining this equation with (12) gives the expression for the hydrogen bond energy in the conformer ZZ

(16) $$E_{\mathrm{HB}}=E^{\mathrm{ZZ}}-\frac{1}{2}\left(E^{\mathrm{EZ}}+E^{\mathrm{EE}}\right)+\Delta_{g}^{\mathrm{av}}$$

but in which there is (so far) the unknown quantity $\Delta_{g}^{\mathrm{av}}$. In fact, the hydrogen bond energy, $E_{\mathrm{HB}}$, and the averaged contribution to the configuration change Z→E, $\Delta_{g}^{\mathrm{av}}$, are formally non-separable quantities. However, the existence of conformers allowed for determining the unknown contribution $\Delta_{g}^{\mathrm{av}}$ from yet another source. Let us introduce the fictitious equivalents of the conformers EZ and EE (EZ${}^{f}$ and EE${}^{f}$, respectively), having the same values of all (of course, except the dihedral angle(s) changing the conformation) geometrical parameters as the conformer ZZ (see Figure 10). The energy that is associated with the transition ZZ${}^{f}\to$EZ can then be assumed in the form

(17) $$\Delta_{g}^{\mathrm{ZZ}\to \mathrm{EZ}}=\Delta^{Z\to E}+\Delta_{\mathrm{rel}}$$

where $\Delta^{Z\to E}$ is the energy resulting from the change of the Z→E configuration while maintaining the constant values of all geometrical parameters, while $\Delta_{\mathrm{rel}}$ is the relaxation energy of the fictitious EZ${}^{f}$ form to its fully relaxed equivalent obtained after the full geometry optimization. The energy associated with the transition ZZ${}^{f}\to$EE can be presented quite similarly

(18) $$\Delta_{g}^{\mathrm{ZZ}\to \mathrm{EE}}={\tilde{\Delta }}^{Z\to E}+{\tilde{\Delta }}_{\mathrm{rel}}$$

Changing the conformation from ZZ${}^{f}$ to either EZ${}^{f}$ or EE${}^{f}$ (i.e., maintaining the same values of bond lengths and angles) should not have a significant influence on the energy change. With the neglect of changing the interactions between ’unbound’ atoms, it can therefore be assumed that $\Delta^{Z\to E}\approx {\tilde{\Delta }}^{Z\to E}\approx 0$. This approximation gives, after adding Equations (17) and (18) to each other, another expression for $\Delta_{g}^{\mathrm{av}}$

(19) $$\Delta_{g}^{\mathrm{av}}=\frac{1}{2}\left(\Delta_{g}^{\mathrm{ZZ}\to \mathrm{EZ}}+\Delta_{g}^{\mathrm{ZZ}\to \mathrm{EE}}\right)\approx \frac{1}{2}\left[\left(E^{\mathrm{EZ},f}-E^{\mathrm{EZ}}\right)+\left(E^{\mathrm{EE},f}-E^{\mathrm{EE}}\right)\right]$$

which, inserted into Equation (16), gives the final formula for the value of the hydrogen bond energy in ZZ-3-aminoacrolein [39]

(20) $$E_{\mathrm{HB}}^{\mathrm{GCM}}=E^{\mathrm{ZZ}}-\frac{1}{2}\left(E^{\mathrm{EZ},f}+E^{\mathrm{EE},f}\right)<0$$

Thus, it can be seen that, to determine $E_{\mathrm{HB}}^{\mathrm{GCM}}$, only the total energy of the fully optimized ZZ conformer and the total energies of the fictitious EZ and EE conformers with the values of geometric parameters (except for the dihedral angle O=C–C=O) from the ZZ conformer are needed. It is worth repeating at this point that GCM, i.e., formula (20), to some extent takes into account the changes in geometric parameters when moving from the ZZ form to the reference forms.

**Figure 11:**
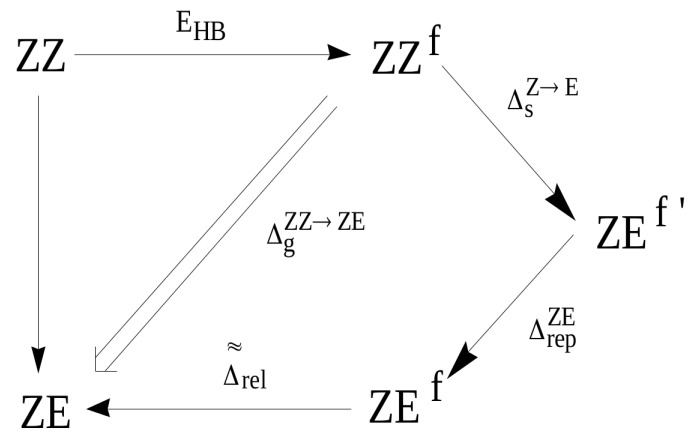
Diagram showing the way of obtaining the ZE conformer from the ZZ one through various fictitious forms.

At this point, it is instructive to compare GCM with the OCM variant, in which the open reference form is the ZE conformer, i.e., $E_{\mathrm{HB}}^{\mathrm{OCM}}=E^{\mathrm{ZZ}}-E^{\mathrm{ZE}}$ (cf. with Equation (4)). As already discussed, the assumption of OCM is that the reference open system does not differ significantly from the closed form, whereas, in the case of ZZ-3-aminoacrolein, the rotation of the aldehyde group around the C–C bond leading to the ZE conformer introduces a new rather significant interaction of the H⋯H type (see Figure 9). This interaction is practically not present in the closed form ZZ. Any estimate that refers to the ZE conformer as the reference form should take this change into account. Suppose (similar to the hydrogen bond in the ZZ conformer) that this H⋯H interaction in the fictitious ZE${}^{f}$ form can be ‘turned off’, which gives ZE${}^{f^{\prime }}$ (note the prime sign in the superscript). The energy associated with the rotation of the aldehyde group ($\Delta_{s}^{Z\to E}$) at the transition ZZ${}^{f}\to$ ZE${}^{f^{\prime }}$ can be assumed to be negligible due to both the conservation of the same geometric parameters as in the conformer ZZ and also due to the neglect of additional H⋯H repulsion at this stage (additionally, the negligible influence of changes in the interactions of unbound atoms other than H⋯H is also assumed). This repulsion leads to an energy increase of $\Delta_{\mathrm{rep}}^{\mathrm{ZE}}$ and to the form ZE${}^{f}$, which still maintains the geometry of ZZ. Only full relaxation of the ZE${}^{f}$ geometry leads to the optimized ZE conformer. The energy that is associated with this relaxation has been designated as ${\tilde{\tilde{\Delta }}}_{\mathrm{rel}}$ (see Figure 11).

Therefore, the energy that is associated with the transition from the ZZ${}^{f}$ conformer to the ZE conformer can be expressed as:

(21) $$\Delta_{g}^{\mathrm{ZZ}\to \mathrm{ZE}}\approx \Delta_{\mathrm{rep}}^{\mathrm{ZE}}+{\tilde{\tilde{\Delta }}}_{\mathrm{rel}}$$

Given the assumption (12) and by the similarity to the previously defined changes in energies $\Delta_{g}^{\mathrm{ZZ}\to \mathrm{EZ}}$ (13) and $\Delta_{g}^{\mathrm{ZZ}\to \mathrm{EE}}$ (14), one gets

(22) $$\Delta_{g}^{\mathrm{ZZ}\to \mathrm{ZE}}=E^{\mathrm{ZE}}-E^{\mathrm{ZZ},f}$$

Inserting this expression together with Equation (21) into Equation (12) gives a relationship between the estimation of the hydrogen bond energy in ZZ-3-aminoacrolein that is obtained by GCM and that obtained by OCM with the ZE conformer as the reference open form

(23) $$E_{\mathrm{HB}}^{\mathrm{GCM}}=E^{\mathrm{ZZ}}-E^{\mathrm{ZE}}+\left(\Delta_{\mathrm{rep}}^{\mathrm{ZE}}+{\tilde{\tilde{\Delta }}}_{\mathrm{rel}}\right)=E_{\mathrm{HB}}^{\mathrm{OCM}}+\left(\Delta_{\mathrm{rep}}^{\mathrm{ZE}}+{\tilde{\tilde{\Delta }}}_{\mathrm{rel}}\right)$$

Equation (23) shows that, when compared to OCM, the estimation that is based on GCM takes into account two terms with opposite signs. The repulsive term $\Delta_{\mathrm{rep}}^{\mathrm{ZE}}$ is positive, whereas the relaxation term ${\tilde{\tilde{\Delta }}}_{\mathrm{rel}}$ is negative. The mutual weights of these two terms cause that the value of the intramolecular hydrogen bond energy determined by GCM is either below or above the value obtained by OCM. Strong hydrogen bonds should cause significant changes within the X-H⋯Y bridge and, thus, both a small distance H⋯Y in the conformer ZZ and a small distance H⋯H in the fictitious form ZE${}^{f^{\prime }}$ (or ZE${}^{f}$) obtained after rotation of the proton-acceptor group while maintaining the geometrical parameters from the conformer ZZ (except for the dihedral angle O=C–C=C). As a consequence, in molecules with a strong intramolecular hydrogen bond, the role of H⋯H repulsion at the ZE${}^{f^{\prime }}\to$ ZE${}^{f}$ should be significant. On the other hand, the significance of the relaxation term ${\tilde{\tilde{\Delta }}}_{\mathrm{rel}}$ should be dominant in the case of relatively small distances H⋯H in the ZE${}^{f}$ form (H⋯Y in ZZ) and, which seems more important, in the case of bulky proton-acceptors.

At this point, it is worth comparing the hydrogen bond energy values that were obtained with GCM with those obtained with the traditional variant of OCM. The first comparison of this type was made for the ZZ-3-aminopropenal (ZZ-3-aminoacrolein) [47] discussed here and for the related ZZ-3-aminopropential [39], where sulfur atom replaces the oxygen atom. The energy values of hydrogen bonds $E_{\mathrm{HB}}^{\mathrm{GCM}}$ and $E_{\mathrm{HB}}^{\mathrm{OCM}}$ are shown in Table 2. Additionally, this table also shows the relative energies (in relation to ZZ) of the respective conformers, the H⋯H distances in ZE${}^{f}$ and ZE forms, and the values of ${\tilde{\tilde{\Delta }}}_{\mathrm{rel}}$, which will be used in the current discussion. All of these values are limited to the best method used (MP2/6-311++G**), so as not to increase the amount of numerical data [39].

In the case of 3-aminoacrolein, the following order of relative energies of conformers was obtained: $E^{\mathrm{ZZ}}<E^{\mathrm{EE}}<E^{\mathrm{EZ}}<E^{\mathrm{ZE}}$. It suggests a significant relaxation of the most extended EE conformer and a significant role of the H⋯H repulsion in the ZE conformer. In the case of 3-aminopropential (Y = S), the relative energy of the EZ conformer, on the other hand, is significantly lifted up, so that it equals that of the ZE conformer. In turn, this result suggests a greater role of S⋯H valence repulsion in 3-aminopropential than O⋯H in the EZ conformer (a complementary explanation may also be the greater role of the attractive component in the O⋯H interaction than S⋯H, which lowers the relative energy of EZ-3-aminoacrolein in relation to EZ-3-aminopropential). It is also manifested by the values of the angle CCY, which is 128.3${}^{\circ }$ and only 125.4${}^{\circ }$ for Y = S and O, respectively. As for the estimated values of the hydrogen bond energy, interestingly, OCM suggests a somewhat stronger N-H⋯O hydrogen bond in ZZ-3-aminoacrolein (−6.50 kcal/mol) than N-H⋯S in ZZ-3-aminopropential (−6.02 kcal/mol), whereas, in the case of GCM, the opposite is obtained, i.e., this method suggests that the latter bond is stronger (−6.96 kcal/mol) than the former one (−5.28 kcal/mol). Table 2 also presents the values of the distances H⋯H in the ZE${}^{f}$ and ZE forms of both molecules, as well as the changes of these distances at the ZE${}^{f}\to$ ZE transition, i.e., upon relaxation of this conformer. The much higher $\Delta d_{H\cdots H}^{\mathrm{ZE},f\to \mathrm{ZE}}$ value for the ZE-3-aminoacrolein (−0.301 Å) than for ZE-3-aminopropential (−0.159 Å) suggests a much stronger H⋯H repulsion in the former of these systems, which is most likely due to the much shorter initial distance (1.840 Å vs. 1.968 Å). This suggestion is actually confirmed by the obtained results. Namely, as can be seen from the last column of Table 2, 3-aminoacrolein and 3-aminopropential are characterized by the same value (−1.87 kcal/mol) of ${\tilde{\tilde{\Delta }}}_{\mathrm{rel}}$, i.e., the relaxation term ZE${}^{f}\to$ ZE. Therefore, the greater change in the H⋯H distance at the transition ZE${}^{f}\to$ ZE for the former of these molecules must result primarily from the greater repulsion $\Delta_{\mathrm{rep}}^{\mathrm{ZE}}$, which, as a consequence, should significantly exceed the relaxation component ${\tilde{\tilde{\Delta }}}_{\mathrm{rel}}$. In turn, this should lead to a significantly lower $E_{\mathrm{HB}}^{\mathrm{GCM}}$ when compared to $E_{\mathrm{HB}}^{\mathrm{OCM}}$. As can be seen from Table 2, such a relationship for ZZ-3-aminoacrolein does indeed take place since $E_{\mathrm{HB}}^{\mathrm{GCM}}$ and $E_{\mathrm{HB}}^{\mathrm{OCM}}$ amount to −5.28 and −6.50 kcal/mol, respectively. This result shows that the hydrogen bond energies obtained within GCM are consistent with the observable geometric changes.

**Figure 12:**
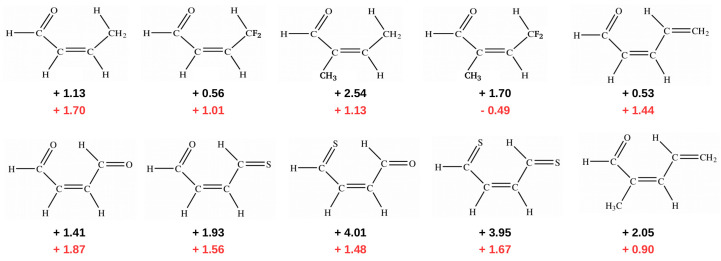
Energy values (in kcal/mol) of intramolecular C-H⋯O/S interactions obtained [41] (B3LYP/aug-cc-pVTZ) by either OCM (**black**) or GCM (**red**).

In addition to the case of 3-aminoacrolein [47] and 3-aminopropential [39] discussed here, GCM was later used to estimate the energy of intramolecular C-H⋯O/S interactions in few systems featuring a similar *quasi*-ring structure (Figure 12) [40,41].

**Figure 13:**
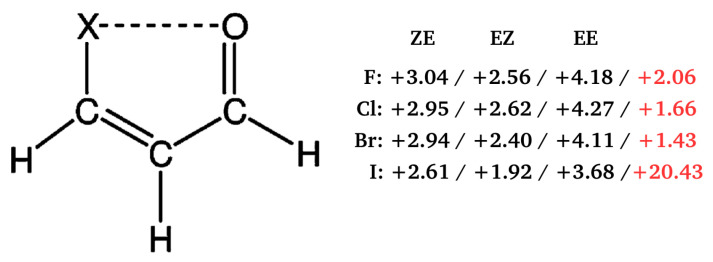
Interaction energies (in kcal/mol) of the X⋯O (X = F, Cl, Br, I) contact obtained [43] by either OCM (**black**) or GCM (**red**). The MP2/aug-cc-pVTZ level of theory was used for all systems but that with Y = I, for which MP2/aug-cc-pVTZ-PP was used instead.

Importantly, contrary to popular belief, these calculations showed that the C-H⋯O/S contacts in these systems are actually destabilizing. Therefore, no hydrogen bond in the usual sense is formed between the proton-donating C-H bond and proton-acceptor O or S atoms. This result was interpreted [40,41] in terms of the steric compression, which leads to the dominance of the valence repulsion contribution in the C-H⋯O contact and it was further supported by observing both the increase in contact destabilization and the corresponding geometric changes during the flattening of some systems. Further detailed studies on an even larger group of systems (*vide infra*) showed, however, that intramolecular C-H⋯O interactions may be destabilizing in some systems, while stabilizing in others [44]. The fact that the large number of X⋯O (X = F, Cl, Br, I), O⋯O and F⋯F interactions, which some consider stabilizing due to the presence of a bond path tracing these contacts are, in fact, destabilizing in many molecules was also shown [43] by means of the energy values obtained, *inter alia*, by GCM and OCM. An example is shown in Figure 13.

Theoretical studies [39,40,41,43,44] show that GCM can be considered to be a reliable method of estimating the energy of both intramolecular hydrogen bonds as well as intramolecular non-bonding interactions. As this method takes into account changes in geometric parameters that occur when passing to reference systems, it is a more reliable approach than the standard OCM, which does not take into account these changes at all. Of course, the applicability of GCM, like most other methods, is limited. For example, the presence of bulky substituents can significantly reduce the reliability of this method. Moreover, of course, the analyzed molecule must have appropriate conformers, which is not always the case. However, OCM also has to deal with similar requirements. Nevertheless, OCM is less tricky.

It is obvious that obtaining the individual conformers needed while using conformational methods requires a great deal of care and attention. Unfortunately, this is not always the case. In their study of the N-H⋯O and N-H⋯S intramolecular hydrogen bonds in β-aminoacrolein, β-thioaminoacrolein, and their halogenated derivatives, Nowroozi and Masumian claimed that GCM performs worse than RBM and RRM, in particular [63]. However, it is enough to look at their Scheme 3 to realize that they used wrong conformers labeled as EZ and EE. Briefly, both of these conformers should have H and R${}_{3}$ at reversed positions! (Starting with the ZZ conformer, rotation of the -NHR${}_{3}$ group around the C=C double bond obviously leaves the H atom rotated with this group on the “inside” of the molecule, i.e., at the R${}_{3}$ site and close to R${}_{1}$.) Because EZ and EE conformers (either real or fictitious) are used in RRM and GCM, it is obvious that the results that are presented by Nowroozi and Masumian [63] are completely wrong (as evidenced, e.g., by low $R^{2}$ values). Moreover, these authors ignored the fact that some of the conformers they used experience new significant interactions, such as O⋯Br, which, of course, significantly affect the total energy of a given conformer.

#### 2.1.5. Geometry-Corrected Related Rotamer Method (GCRRM)

It is worth noting that, when compared to GCM, RRM should give too negative values of interaction energy, because the total energy of the ZE conformer, $E^{\mathrm{ZE}}$, which is not present in the formula for $E_{\mathrm{HB}}^{\mathrm{GCM}}$ (Equation (20)), appears with a negative sign. At first glance, it would seem difficult to further directly compare the two methods, as they do not use the same EZ and EE conformer structures; GCM overlays them with the values of the geometric parameters from the closed ZZ form, while RRM uses fully relaxed geometries. Nevertheless, the difference in estimations of the two methods can be written, as follows [44]:

(24) $$E_{\mathrm{HB}}^{\mathrm{GCM}}-E_{\mathrm{HB}}^{\mathrm{RRM}}=\frac{1}{2}\left(E^{\mathrm{EZ}}-E^{\mathrm{EZ},f}\right)+\frac{1}{2}\left(E^{\mathrm{EE}}-E^{\mathrm{EE},f}\right)+\frac{1}{2}\left(E^{\mathrm{EZ}}-E^{\mathrm{EE}}\right)+\left(E^{\mathrm{ZE}}-E^{\mathrm{EE}}\right)$$

This expression shows that the difference between the estimates that were obtained with GCM and RRM results from the balance of the relaxation terms (first two terms) and the conformational changes (EZ→EE and ZE→EE). Importantly, both of these contributions have opposite signs and the latter ones are larger than the former. Moreover, the last term contributes without the factor 1/2. As a consequence, the difference (24) is positive. In the case of 13 molecules containing intramolecular C-H⋯O contacts considered in the reference [44], the energies of the consecutive terms were, as follows: −1.3 ± 0.3, −2.0 ± 0.6, 2.5 ± 0.8, and 2.7 ± 0.3 kcal/mol, so that the difference (24) was 2.3 ± 0.3 kcal/mol. Because the first three values almost cancel themselves (−0.4 kcal/mol), it can be assumed that the difference (24) comes mainly from the configurational change EE→ZE. This configurational change can then be considered as a two-step process: EE→EE${}^{f^{\prime }}\to$ZE, where EE${}^{f^{\prime }}$ is a fictitious conformer EE having the geometric parameters of the ZE conformer. Hence, the energy of the EE→ZE process can be written as the sum of the preparation energy of the ZE conformer and the E→Z isomerization energy:

(25) $$E^{\mathrm{ZE}}-E^{\mathrm{EE}}=\left(E^{\mathrm{EE},f^{\prime }}-E^{\mathrm{EE}}\right)+\left(E^{\mathrm{ZE}}-E^{\mathrm{EE},f^{\prime }}\right)$$

**Figure 14:**
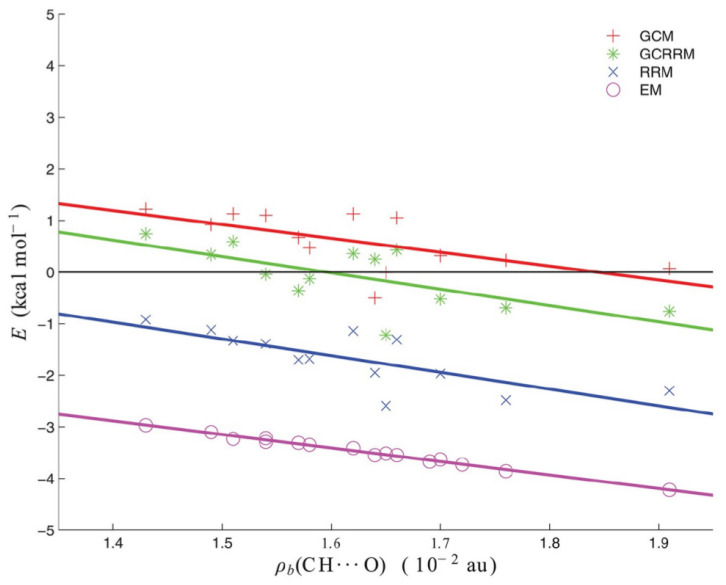
Interaction energies (in kcal/mol) of the intramolecular C-H⋯O contacts in the molecules investigated in ref. [44].

In the considered systems with the intramolecular C-H⋯O interactions, the first term was 1.0 ± 0.2 kcal/mol. The second term is related to the H⋯H repulsion that appears in the conformer ZE, the value of which was estimated at 0.60 ± 0.17 kcal/mol (median value) [44]. Together with the preparation energy, this energy suggests that the EE→ZE process is affected by close H⋯H contact by roughly 1.6 kcal/mol, which is close to the actual value of 2.7 kcal/mol as well as the E→Z isomerization energy in 2-butene (1.04 kcal/mol). This fairly good agreement led to the proposition of a corrected RRM known as the Geometry Corrected Related Rotamers Method (GCRRM) [44]. According to GCRRM, the estimated value of an intramolecular hydrogen bond (or other interaction) can be obtained from the following formula

(26) $$E_{\mathrm{HB}}^{\mathrm{GCRRM}}=E_{\mathrm{HB}}^{\mathrm{RRM}}+\left(E^{\mathrm{EE},f^{\prime }}-E^{\mathrm{EE}}\right)+E_{\mathrm{HH}}$$

where the $E^{\mathrm{HH}}$ value is 0.6 kcal/mol. The values obtained with GCRRM are between the values obtained with GCM and RRM and closer to the former, as shown in Figure 14.

It is noteworthy that all four lines that are shown in Figure 14 have similar slopes; therefore, the methods differ by their intercepts that can be seen as “zeros of the interaction energy” [44]. Indeed, $E_{\mathrm{HB}}^{\mathrm{RRM}}=E_{\mathrm{HB}}^{\mathrm{EM}}+1.7$ kcal/mol, $E_{\mathrm{HB}}^{\mathrm{GCRRM}}=E_{\mathrm{HB}}^{\mathrm{EM}}+3.4$ kcal/mol and $E_{\mathrm{HB}}^{\mathrm{GCM}}=E_{\mathrm{HB}}^{\mathrm{EM}}+4.0$ kcal/mol. At the same time, Figure 14 is a wonderful illustration displaying that a given intramolecular interaction in a certain system may be suggested to be much or less stabilizing according to one estimating method while another method may suggest its rather repulsive nature.

### 2.2. Rotation Barriers Method (RBM)

**Figure 15:**
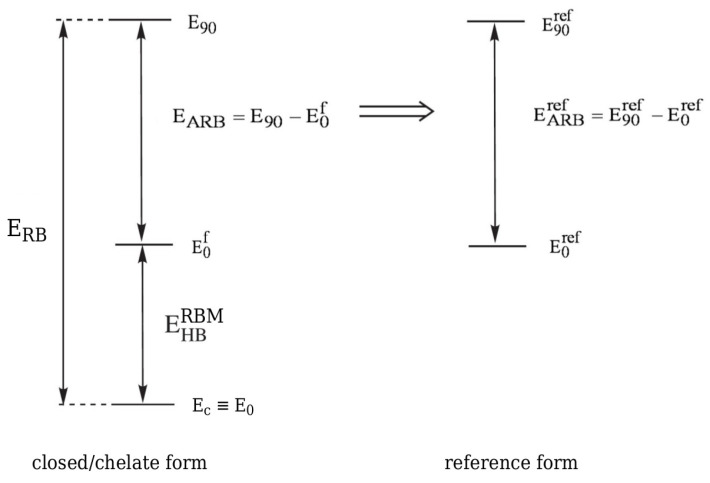
Scheme showing the way of estimating the energy of an intramolecular hydrogen bond according to RBM.

A strong alternative to OCM with its various variants is the Rotation Barriers Method (RBM) [33,65,66,67,68,69,70,71,72] first used by Buemi et al. in order to estimate the energy of the O-H⋯O intramolecular hydrogen bond in malonaldehyde [66] and a bit later of N-H⋯N in formazan [67]. Quite rightly, this method assumes that an intramolecular hydrogen bond in the closed (chelate) form raises the height of the energy barrier that is associated with either the proton-donor or proton-acceptor group rotation by 180${}^{\circ }$ to form an open form. Hence, when assuming the additivity of the respective energy terms, it can be written that

(27) $$E_{\mathrm{RB}}=E_{\mathrm{HB}}^{\mathrm{RBM}}+E_{\mathrm{ARB}}$$

where $E_{\mathrm{RB}}$ is the rotation barrier and $E_{\mathrm{ARB}}$ is (to use Buemi’s terminology) the actual rotation barrier of the considered group [33]. The actual rotation barrier introduced as a result of the above additivity scheme is obviously related to a fictitious equivalent of a closed system in which the intramolecular hydrogen bond is ‘turned off’, and, therefore, it is not possible to calculate its value exactly. Nevertheless, $E_{\mathrm{ARB}}$ can be estimated while using a certain reference system (see Figure 15).

Hence,

(28) $$E_{\mathrm{HB}}^{\mathrm{RBM}}=E_{\mathrm{ARB}}^{\mathrm{ref}}-E_{\mathrm{RB}}=\left(E_{90}^{\mathrm{ref}}-E_{0}^{\mathrm{ref}}\right)-\left(E_{90}-E_{c}\right)<0$$

where the expressions in the former and in the latter brackets are rotation energy barriers for either the proton-donor or the proton-acceptor group in the reference and the closed form, respectively. In fact, the transition states for the rotations in both systems do not have to correspond exactly to the perpendicular orientation of the group. Nevertheless, the symbols denoting total energies of the transition states are given the subscript 90 in order to emphasize that often the transition state, that is associated with the rotation of a given group, roughly corresponds to its perpendicular orientation with respect to the molecular framework. Importantly, just like in the case of OCM, in RBM it is assumed that the reference system retains the earlier described significant similarity to the bound, i.e., closed form. This condition is not always easy to meet. On the other hand, the use of RBM is a reasonable method of choice in many of those cases where the energy estimate based on OCM is unreliable due to the presence of some bulky or highly electronegative substituents leading to new important interactions in the open reference form [33].

**Figure 16:**
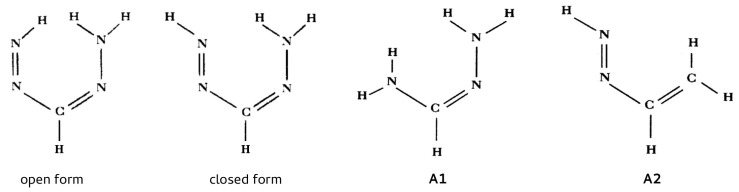
The open and closed forms of formazan and the two reference systems (**A1** and **A2**) used by Buemi et al. [67] in RBM.

As already mentioned, this method was first used by Buemi et al. [67] in order to estimate the energy of the N-H⋯N intramolecular hydrogen bond in one of the conformers of formazan (Figure 16).

The abandonment of the traditional OCM and the need to use a different method, which led to RBM, resulted from the inability to find a reliable reference form. Because of the symmetry of the amino group, its rotation is useless, and the rotation of the N=N–H group leads to a close H⋯H contact. On the other hand, the use of other conformers was considered [67] impractical, because it led to too large structural change. As a consequence of these problems, Buemi et al. proposed using, in RBM, two reference systems shown in Figure 16 as A1 and A2. Buemi et al. emphasized that they had previously successfully used this method to determine the interaction energy of the O-H⋯O intramolecular hydrogen bond in malonaldehyde (using vinyl alcohol as a reference), obtaining (MP2/6-31G**) a value similar to that of the traditional open-closed method (−14.07 and −14.01 kcal/mol, respectively) [66]. Depending on the reference molecule A1 or A2 and on more subtle conditions concerning the structure of the amino group (planar vs. pyramidal), Buemi et al. obtained energies that ranged from −9.38 to −4.85 kcal/mol. Subsequently, however, the value close to the middle, i.e., −7.17 kcal/mol, was considered as the most reliable. Nevertheless, quite reasonably, Buemi and Zuccarello pointed out that such wide range of the obtained estimates does not allow for stating that the estimate of the N-H⋯N hydrogen bond energy in formazan is as good as O-H⋯O in malonaldehyde [66].

**Figure 17:**
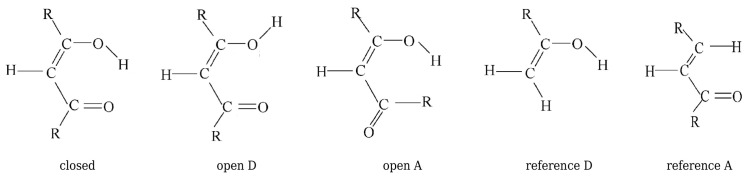
The closed form, the two open forms and the two reference molecules used in RBM for malondialdehyde and acetylacetone (R = CH_3_) [33].

Buemi and Zuccarello then used RBM to estimate interaction energies of various intramolecular hydrogen bonds (O-H⋯O, O-H⋯halogen, O-H⋯N, N-H⋯O, N-H⋯N, S-H⋯O, O-H⋯S, and S-H⋯S) in many molecules (e.g., malondialdehyde, acetylacetone, and their variously substituted derivatives, formazan, 3-aminoacrolein, some β-thioxo- and β-dithioketones, 2-halophenols, 2-nitrophenol) [33]. From the many data shown there, I will only mention those obtained for malondialdehyde, acetylacetone, and 3-aminoacrolein. The closed form, the two open forms, and the two reference molecules used in RBM are shown in Figure 17, and the quoted values of the respective estimates are listed in Table 3.

As can be seen from Table 3, in the RBM calculations, Buemi and Zuccarello used four reference structures, two for the ARB for the -OH proton-donor group, and two for the ARB for the proton-acceptor -CHO group. In the former case, these systems were the open form A and the reference D obtained by replacing the group -CHO by the H atom. In the latter case, these were the open form D and the reference A obtained by replacing the OH group by H. Buemi and Zuccarello emphasized the very good agreement of the estimates based on OCM and RBM, whenever these methods use the proton-donor group rotation [33]. This result is especially obvious in the case of malondialdehyde (ca. −14 kcal/mol), whereas slightly less in the case of acetylacetone (from ca. −17 kcal/mol to ca. −15 kcal/mol), which was attributed to the new, probably quite significant, interaction between the methyl group and the hydrogen atom from the hydroxyl group in the open form A. On the contrary, worse agreement of the OCM and RBM results was noted for the estimates that are based on the rotation of the proton-acceptor group. However, it is noted that, in general, the estimates that are based on RBM (no matter whether it is a rotation of the proton-donor or the proton-acceptor group) are closer to OCM estimates based on the proton-donor group rotation than the corresponding OCM based on the proton-acceptor group rotation [33].

**Figure 18:**
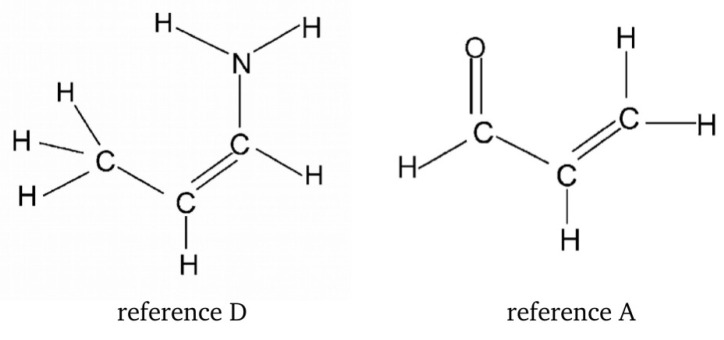
Two reference molecules used in RBM for 3-aminoacrolein [33].

As already mentioned, for ZZ-3-aminoacrolein (see Figure 9), it seems that the most reasonable reference form is EZ (although Buemi and Zuccarello also admitted the conformer ZE, this form experiences a new significant H⋯H interaction). In the case of malonaldehyde, the EZ conformer gives a value of −9.7 kcal/mol, thus approximately 5.3 kcal/mol lower than the classic value of the O-H⋯O hydrogen bond energy in malondialdehyde. Assuming that a similar underestimation would also act for 3-aminoacrolein, Buemi and Zuccarello renormalized the obtained value (−5.2 kcal/mol), finally obtaining a value of about −10.5 kcal/mol [33]. The main model problem in the estimation of the N-H⋯O hydrogen bond energy in ZZ-3-aminoacrolein using RBM is the change in the degree of amino group pyramidalization during rotation [33,47]. Because of the presence of the hydrogen bond, this group is planar in the ZZ conformer, whereas slightly pyramidal when rotating around the C-N bond. Depending on the constraint put on the rotating amino group and the reference system utilized (Figure 18), the estimated value of the N-H⋯O hydrogen bond energy in ZZ-3-aminoacrolein is between −11.7 and −8.4 kcal/mol (MP2/6-31G**).

Unfortunately, this example shows quite a lot of freedom in terms of the possible choice of reference systems. On the one hand, the reference molecule A was obtained for ZZ-3-aminoacrolein by replacing the amino group with a hydrogen atom, whereas molecule D by replacing the aldehyde group with a methyl group (and not only with hydrogen). On the other hand, both of these reference molecules have the same number of heavy framework atoms. However, unlike D, molecule A features the presence of a conjugated system of two double bonds. Hence, it should be expected that the π-electron structure in both of these reference molecules is quite different.

In summary, RBM is a reasonable approach for estimating intramolecular hydrogen bond energy in many simple molecules and it can be successfully used as a replacement or supplement to the estimation based on OCM. However, like OCM, this method should also be used with great caution, because the presence of new interactions during rotation of a group in the parent or reference molecule may significantly reduce the reliability of the estimation. Moreover, in this method, both the problem of choosing a reasonable reference form and a certain freedom of this choice are noticeable. As noted by Buemi and Zuccarello [33], RBM is much more computationally expensive than OCM, as it requires calculating the rotation barriers for two systems, the bound, i.e., the closed one, and the reference molecule (Equation (28)). It is worth reminding here that the maxima of these barriers do not have to correspond exactly to the perpendicular arrangement of the rotated groups.

### 2.3. Dimer Model (DM)

Importantly, in the context of the present considerations, all of the methods of estimating the energy of intramolecular hydrogen bond (or more generally, interaction) in the closed form that were discussed so far, were based on the assumed model of energy additivity, which leads to quite a lot of freedom in choosing a reasonable reference system. This, in turn, leads to the known problem that the resulting hydrogen bond energy value can be quite dependent on this reference system. Moreover, even within the adopted estimation method there are often many possible variants (e.g., in OCM with only partial optimization of the open reference form). Hence, the idea was born to abandon the assumed total energy partition of the closed form and refer to the strictly defined interaction energy of the *inter*molecular contact (Equation (1)). This idea leads to the Dimer Model (DM) [73,74]. This model was most likely used for the first time by Palusiak and Krygowski in order to estimate the interaction energy of the intramolecular π⋯π contact in 1,3,5,7-cyclooctatetraene [73]. Subsequently, DM was used by Jabłoński and Palusiak [74] to support the previously obtained result [43] that the intramolecular Cl⋯O interaction in 3-chloropropenal is, in fact, destabilizing (repulsive) and not stabilizing [75].

**Figure 19:**
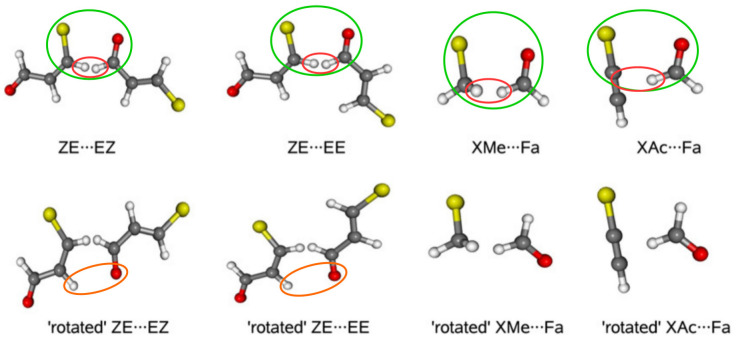
Spatial arrangement of reference dimers and their ‘open’ forms used in estimating the interaction energies of intramolecular X⋯O contacts in ZZ-3-halogenopropenal [74].

To relate to the results that were obtained for the 3-halogenopropenal previously presented in Figure 13, details on the ideas of DM will be discussed on the basis of this molecule [74]. The first step in this model is to build a reasonable dimer in which the fragment of the considered interaction from the bound molecule, i.e., its closed form, is preserved. In the case of 3-halogenopropenal, this is obviously the C–X⋯O=CH fragment in the ZZ conformer (see Figure 13). In order to test the reliability of the model, two dimers, namely ZE⋯EZ and ZE⋯EE, were constructed where, fundamentally, the C–X⋯O=CH fragment was taken from the ZZ-3-halogenopropenal and then built into these dimers, as shown by green ovals in Figure 19.

Unfortunately, as clearly seen (red ovals) in Figure 19, the new very short C-H⋯H-C contacts appear in the dimers thus constructed. However, they can be accounted for (and ‘subtracted’) by using appropriate rotated (inverted) forms of the proposed dimers, in which, importantly, the relative arrangement of all atoms in the C-H⋯H-C fragment is conserved. Consequently, the formula for the interaction energy of the intramolecular X⋯O contact in ZZ-3-halogenopropenal has the following form:

(29) $$E_{X\cdots O}^{\mathrm{DM}}=E_{\mathrm{int}}\left(\mathrm{dimer}\right)-E_{\mathrm{int}}\left(\mathrm{rotated}\;\mathrm{dimer}\right)$$

Table 4 presents the results obtained using this formula.

First, it should be noted that the thus obtained estimates are positive, not negative. This result confirmed the previously [43] obtained conclusion that the intramolecular X⋯O interactions in ZZ-3-halogenopropenal are in fact locally destabilizing, i.e., repulsive. As expected, the obtained repulsion values increase in the order F < Cl < Br, and those obtained for Cl and Br are similar to each other, whereas the values for F differ from them. Due to a probably slight (2.44 Å) contamination of the rotated dimers with weak C-H⋯O hydrogen bond (orange ovals), simplification of DM was then applied. Namely, halogenomethane⋯formaldehyde (XMe⋯Fa) and halogenacetylene⋯formaldehyde (XAc⋯Fa) dimers were then designed (Figure 19) with imposed restrictions on the structural requirements discussed earlier [74]. Although these simplified variants introduce some subtle problems [74] and the resulting estimates are clearly lower, the values are still positive, which supports the earlier conclusion regarding the repulsive nature of the X⋯O contact in ZZ-3-halogenopropenal.

### 2.4. Isodesmic Reactions Method (IRM)

In many areas of physical organic and theoretical chemistry the so-called isodesmic reactions are used [63,76,77,78,79,80,81,82,83,84,85,86,87,88,89,90,91,92,93]. These are more or less hypothetical reactions, in which the same numbers of single and multiple bonds of the same type are present on both sides of this reaction, i.e., of the reagents and of the products. If, in addition, the relevant atoms conserve their hybridization, then these reactions are called the homodesmotic reactions [79,80,81,82,83,87,88,89,90,91,92]. The conservation of the atomic hybridizations makes the homodesmotic reaction a more reliable description of a given phenomenon than the less demanding isodesmic reaction. The use of isodesmic and homodesmotic reactions allowed for a more detailed theoretical description of many physical processes and effects, such as the extra stability due to cyclic π-electron delocalization [88]. Homodesmotic reactions are also often used in order to estimate the energy of intramolecular hydrogen bonds [32,38,39,44,63,84,86,87,89,91] or some other interactions of interest [42,43,44,55,87,93].

**Figure 20:**
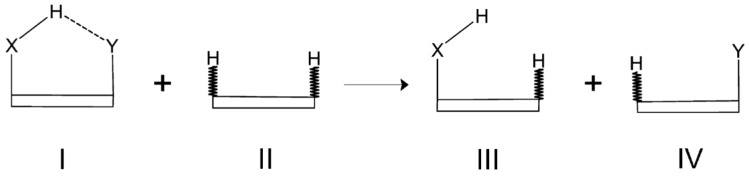
General scheme showing a homodesmotic reaction for a model molecule featuring an intramolecular X-H⋯Y hydrogen bond.

The reliability of the Isodesmic Reactions Method (IRM) is based on the assumption that the total energy of a molecule I can be partitioned into energies of chemically recognizable fragments, such as bond energies, and that those energies are transferable among various molecules which, however, involve similar chemical units. A general scheme of a simple homodesmotic (also isodesmic) reaction for a model system featuring an intramolecular X-H⋯Y hydrogen bond is shown in Figure 20.

In this figure, the molecular framework, which, of course, may vary from molecule to molecule, is drawn as a box for simplicity and, moreover, those C-H bonds in molecules II, III, and IV, which are not present in the parent molecule I are marked by a zigzag bond line. When comparing both sides of the homodesmotic reaction shown in Figure 20, it can be easily seen that all the bonds, except the only one denoting the H⋯Y contact in the parent molecule I, on the left side of this reaction are also present on the right side of this reaction. Accordingly, the only missing ’bond’ on the right side of this reaction equation is the intramolecular H⋯Y contact in the parent molecule I. Thus, the interaction energy of this contact can be obtained by the following expression

(30) $$E_{\mathrm{HB}}^{\mathrm{IRM}}=E\left(I\right)-E^{f}\left(I\right)<0$$

where

(31) $$E^{f}\left(I\right)=E\left(\mathrm{III}\right)+E\left(\mathrm{IV}\right)-E\left(\mathrm{II}\right)$$

In these two equations, *E*(I) is the total energy of the fully optimized parent molecule I, whereas $E^{f}$(I) can be regarded to as the total energy of its fictitious counterpart featuring no H⋯Y contact.

As with conformational methods, the question now arises as to what geometries to use for the auxiliary molecules II, III, and IV [42,43,44,91]. The vast majority of calculations that are related to isodesmic or homodesmotic reactions use fully optimized geometries, so that the total energies in Equations (30) and (31) are total energies of fully optimized molecules. For this reason, such an approach can lead to considerable doubts regarding the reliability of $E_{\mathrm{HB}}^{\mathrm{IRM}}$ if only full geometry optimization of at least one of the molecules leads to new significant interaction(s) or to a significant change in molecular structure compared to I [42]. On the other hand, if the structural fragments in II, III, and IV do not differ significantly from those in I, then IRM can give reasonable estimates of interaction energies of the hydrogen bond (or any other contact of interest) in I. It seems that rigid ring molecules should be privileged here [44]. Another possibility that is very rarely considered [42,43,44] is that the geometry of the parent molecule I is transferred to the auxiliary molecules II, III, and IV. However, then, the question arises, what to do with the C-H bonds that the molecule I does not have (they are indicated in Figure 20 by a zigzag line). Hence, a field for different IRM variants arises here. For example, these bonds can be optimized, or they can be given the length of either the C-X or C-Y bond of molecule I, or any other reasonable value as, e.g., 1 Å. It is worth mentioning that the use of not fully optimized molecules II, III, and IV leads to an overestimation of the hydrogen bond energy value in I, i.e., $E_{\mathrm{HB}}^{\mathrm{IRM}}$.

**Figure 21:**
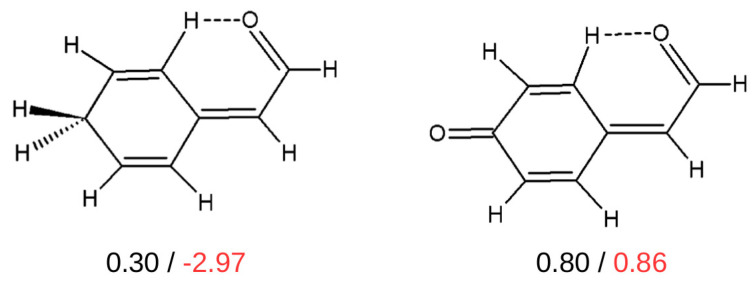
The values (in kcal/mol) of the interaction energies of the C-H⋯O contacts estimated by OCM (black values) and IRM (red values) [44].

Despite the fact that, as mentioned above, the method of estimating the interaction energy either of some hydrogen bonds or some other kinds of intramolecular interactions in I that is based on isodesmic/homodesmotic reactions is quite popular [32,38,39,42,43,44,55,63,84,86,87,89,91,93], its reliability, in general, may raise some doubts. For example, it has been shown that the comparison of the interaction energy in closely related systems disqualifies IRM (Figure 21) [44].

**Figure 22:**
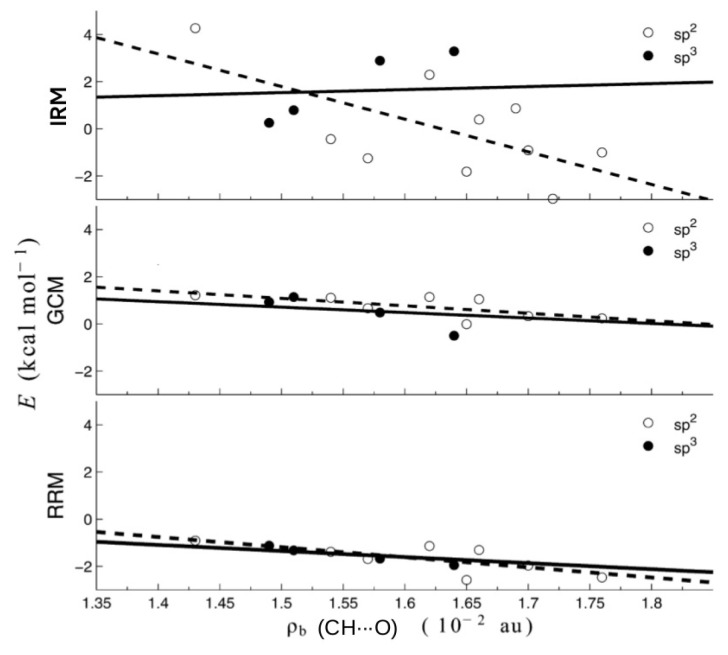
Correlations between RRM-, GCM-, and IRM-based interaction energies of C-H⋯O contacts featuring either sp^2^ (open circle) or sp^3^ (full circle) hybridized carbon atoms and the electron density at the bond critical points of these contacts [44].

As one can see, the values of the interaction energies of C-H⋯O contacts in both of these similar molecules are very close to each other (0.30 and 0.80 kcal/mol) when OCM is used, while IRM gives values very different from each other, also in terms of sign (−2.97 and 0.86 kcal/mol). Moreover, the failure of IRM also manifested itself in the unphysical positive slope of the dependence of $E_{\mathrm{HB}}^{\mathrm{IRM}}$ on the electron density at the critical point ($\rho_{b}$) of the C-H⋯O interaction featuring a sp^3^ hybridized carbon atom (see Figure 22) [44].

In contrast, RRM and GCM gave physically justifiable negative slopes.

### 2.5. QTAIM-Based Methods

By operating with the topology of the electron density distribution, the Quantum Theory of Atoms in Molecules (QTAIM) created by Bader [94,95,96] makes it possible to divide the space of a molecule into closely adjoining, i.e., non-overlapping, three-dimensional subspaces. According to QTAIM, they are understood as individual atoms. These atoms are separarated from each other by surfaces through which there is no electron density gradient flow, and atomic nuclei are most often attractors of this gradient field. Importantly, the possibility of unequivocally defining the space of an atom in a molecule, introduced by QTAIM, enables the determination of many atomic quantities by integration over the volume of a given atom. Yet another useful result of QTAIM are the concepts of the so-called bond path (BP) and bond critical point (BCP) [94,95,96]. The latter is a saddle point in the electron density distribution that features positive curvature (thus, experiencing the minimum) in the direction of the two neighboring nuclei and negative curvatures (thus, experiencing maxima) in the two perpendicular directions, and the former is a pair of gradient vector paths originating at this BCP and terminating at both nuclei. Thus, a bond path is also a ridge of the highest electron density between a pair of the so linked atoms. For this reason, the pattern of such electron density ridges, i.e., bond paths (the so-called molecular graph) very often corresponds to the pattern of bonds that are drawn by chemists, i.e., the structural formula [97]. The fundamental issue in the theory of inter- and intramolecular hydrogen bonds or other stabilizing interactions is that, according to QTAIM [94,95,96], the simultaneous presence of a bond path and a bond critical point between any pair of atoms is a necessary and sufficient proof that this bond (interaction) is stabilizing [98]. This view has resulted in many people using the presence of a bond path as a sufficient criterion for the presence of a hydrogen bond (or other bonding interaction) between a pair of atoms. Unfortunately, this happens without even specifying the bonding (interaction) energy value, in a way ignoring the fact that the hydrogen bond must be stabilizing to deserve this name. However, it should be noted that the treatment of both BP and BCP as sufficient evidence for stabilizing interaction has been criticized [99,100,101,102,103,104,105,106,107,108,109,110,111,112,113,114], as it turns out that the presence of these topological features between a pair of atoms in general does not determine the stabilizing or destabilizing nature of the interaction between the pair [112,113].

However, in the context of this article, it is more important that QTAIM makes it possible to determine the interatomic interaction energy. The first of these methods was proposed by Espinosa et al. [115] and it is based on a quantity computed at the bond critial point of the interaction, the energy of which is to be determined. The another method is based on the partition of the total energy of a system into individual monoatomic and diatomic contributions and functions under the name of Interacting Quantum Atoms (IQA) [116,117]. These two methods will now be discussed in the next two subsections.

#### 2.5.1. Espinosa’s Method (EM)

Based on empirical data for many systems featuring intermolecular hydrogen bonds of the H⋯O type, Espinosa et al. [115] proposed the following relationship between the energy of an intermolecular hydrogen bond and the local electronic potential energy density that is determined at the bond critical point of a given hydrogen bond

(32) $$E_{\mathrm{HB}}^{\mathrm{EM}}=\frac{1}{2}V_{\mathrm{BCP}}$$

It should be noted that both the simplicity of the formula (32) and the easy availability of the $V_{\mathrm{BCP}}$ value (QTAIM calculations) resulted in a rather uncritical acceptance of this expression for determining not only the energy of intermolecular hydrogen bonds, but also the energy of intramolecular interactions. The latter, however, must be firmly criticized [44]. Since $V_{\mathrm{BCP}}$ is a negatively defined quantity [94], the energy of any hydrogen bond (or other interaction) determined by formula (32) will always give a negative $E_{\mathrm{HB}}^{\mathrm{EM}}$ value. Therefore, according to EM, any interaction will be stabilizing. However, as shown [44], many C-H⋯O contacts, which many would probably consider weak hydrogen bonds, are, in fact, destabilizing, i.e., repulsive in nature. Indeed, $V_{\mathrm{BCP}}$, which is crucial in formula (32), was interpreted [115] as the pressure exerted by the system on the electrons in the closest vicinity of BCP. Therefore, it is easy to imagine a situation that the short distance H⋯Y is merely forced, e.g., by the stiffness of the molecular skeleton or some steric interactions, which lead to a high value of $V_{\mathrm{BCP}}$ and, consequently, to a large value of $E_{\mathrm{HB}}^{\mathrm{EM}}$, suggesting strong hydrogen bond, though in reality the interaction may be locally repulsive in nature. For this reason, the use of EM in the cases of intramolecular interactions is not recommended [44].

It should be added that some concerns regarding EM have also been pointed out by Gatti et al. [118] and more recently by Nikolaienko et al. [119]. For example, the latter authors complained that the expression (32) was obtained while using data relating to crystallographic structures in which, as is known, the distances H⋯Y are often much shorter due to lattice forces. Moreover, this expression was obtained for X-H⋯O (X = C, N, O) hydrogen bonds only and its use for hydrogen bonds of other types is unreliable. They also refer to the example of hydrogen bonds of the H⋯F type, for which the Espinosa formula (32) should rather have a factor of 0.31 [120]. To all of these allegations [119], it can be added that the hydrogen bond energies were obtained [115] with a real mixture of theoretical methods. Therefore, it seems necessary to revise the derivation of formula (32). In fact, some modifications to the orginal Espinosa’s formula (Equation (32)) have been proposed [44,118,120,121]. For example, Afonin et al. [121] obtained the formula

(33) $$E_{\mathrm{HB}}=0.277V_{\mathrm{BCP}}+0.45$$

in which the slope value of 0.277 is very close to the mentioned 0.31 value that was obtained by Mata et al. [120]. Even a little earlier, Jabłoński and Monaco proposed correcting the Espinosa’s formula (32) by adding a constant *k* of 3.4 kcal/mol, thus to use the expression $E_{\mathrm{HB}}=E_{\mathrm{HB}}^{\mathrm{EM}}+3.4$ [44]. Importantly, this expression was specifically dedicated to intramolecular hydrogen bonds.

#### 2.5.2. Interacting Quantum Atoms (IQA)

As already mentioned, the Interacting Quantum Atoms (IQA) approach [116,117], which is based on QTAIM, allows for the total energy of a system to be divided into mono- and polyatomic components. Among many energy terms available by means of IQA, the most important one in the context of this article is the interatomic interaction energy defined as follows

(34) $$E_{\mathrm{int}}^{E_{1}E_{2}}=V_{\mathrm{nn}}^{E_{1}E_{2}}+V_{\mathrm{en}}^{E_{1}E_{2}}+V_{\mathrm{ne}}^{E_{1}E_{2}}+V_{\mathrm{ee}}^{E_{1}E_{2}}\;\;\;\;\;\;\;\;\;\left(E_{1}\neq E_{2}\right)$$

where $V_{\mathrm{nn}}^{E_{1}E_{2}}$ is the repulsion energy between nuclei of atoms E_1_ and E_2_, $V_{\mathrm{en}}^{E_{1}E_{2}}$ is the attraction energy between electrons of the atom E${}_{1}$ and the nucleus of the atom E_2_, $V_{\mathrm{ne}}^{E_{1}E_{2}}$ is the attraction energy between the nucleus of the atom E${}_{1}$ and the electrons of the atom E_2_, and $V_{\mathrm{ee}}^{E_{1}E_{2}}$ is the interatomic two-electron repulsion energy. Because the $E_{1}$ and E_2_ atoms may be e.g., the H and Y atoms from the X-H⋯Y hydrogen bridge, it is evident that IQA via Equation (34) can be a suitable tool for calculating the energy of inter- and, more importantly, intramolecular hydrogen bonds. In this case, Equation (34) takes the following form

(35) $$E_{\mathrm{int}}^{H\cdots Y}=E_{\mathrm{HB}}^{\mathrm{IQA}}=V_{\mathrm{nn}}^{\mathrm{HY}}+V_{\mathrm{en}}^{\mathrm{HY}}+V_{\mathrm{ne}}^{\mathrm{HY}}+V_{\mathrm{ee}}^{\mathrm{HY}}$$

It should be emphasized that the determination of the interaction energy of H⋯Y using formula (35) does not require assuming any reference system or referring to empirical data, and from this point of view the IQA-based approach is absolutely unique and, therefore, also worth a wider study of its applicability. It should also be added that $E_{1}$ and $E_{2}$ of Equation (34) can be any atoms, and therefore the interaction energy of any interatomic contact, not just hydrogen bonding, can be determined in a similar way. Moreover, these atoms do not need to be linked to each other by a bond path, nor do they need be in a close proximity to each other.

Unfortunately, as compared to intermolecular hydrogen bonds [122,123,124,125,126], the IQA-based estimates of the energy of intramolecular hydrogen bonds are relatively rare [127,128,129,130]. It is worth noting here that the list of IQA applications given most recently by Guevara-Vela et al. [117] can be easily supplemented with various repulsive interactions [105,106,107,108,112,113,114], which are often related to the presence of an appropriate bond path. Therefore, these cases are important in the very discussion on the interpretation of a bond path and the earlier connection of its presence on molecular graphs with the stabilizing nature of interactions, as stated in the orthodox QTAIM [94].

It should be emphasized that, as it seems, the interatomic interaction energy itself (i.e., $E_{\mathrm{int}}^{E_{1}E_{2}}$) is currently not as popular quantity as the exchange-correlation component ($E_{\mathrm{ee},\mathrm{xc}}^{E_{1}E_{2}}$) of the interelectron interaction energy ($E_{\mathrm{ee}}^{E_{1}E_{2}}=E_{\mathrm{ee},C}^{E_{1}E_{2}}+E_{\mathrm{ee},\mathrm{xc}}^{E_{1}E_{2}}$). This, in turn, results from the fact that $E_{\mathrm{ee},\mathrm{xc}}^{E_{1}E_{2}}$ was associated with the presence of bond path via the concept of so-called privileged exchange channels [131]. Moreover, even more importantly, at short distances [113]$E_{\mathrm{ee},\mathrm{xc}}^{E_{1}E_{2}}$ is related to the strength of a given bond [123]. Therefore, it turns out that, despite the possibility of determining the interatomic interaction energy and thus also of an intramolecular hydrogen bond, which is significant for the theory of intramolecular interactions, this quantity in IQA has become less important than dimensionally much lower exchange energy.

### 2.6. Empirically-Based Methods

It is well known that, apart from the energy or ethalphy of hydrogen bond, i.e., the quantities that directly prove its stabilizing nature, there is a whole range of quantities that indicate or rather suggest the presence of a hydrogen bond indirectly [5]. The typical effects that are to prove the presence of (strong) standard (i.e., those where both X and Y atoms in the X-H⋯Y contact are strongly electronegative) hydrogen bonds include an elongation of the proton-donor X-H bond, shifting the frequency of its stretching vibration towards lower values (i.e., the so-called red-shift) [132,133,134,135], intensification and broadening of the band associated with this vibration [136,137], and deshilding of the proton participating in the hydrogen bond [138,139,140,141] in the magnetic field [142,143] observed in the ^1^H NMR spectra. While all of these effects can be relatively easily correlated with the hydrogen bond energy in the case of intermolecular hydrogen bonds, which, of course, is due to the relatively simple availability of the hydrogen bond energy (or enthalpy of formation) in such a case, transferring these correlations to the ground of intramolecular hydrogen bonds [144,145,146,147,148,149] is much more troublesome. Obviously, this, in turn, results from both the lack of an unambiguous definition of the interaction energy of intramolecular hydrogen bonds and the problematic determination of an unperturbed reference value. Moreover, the rationale for such transferability is unclear and certainly deserves to be a hotly debated topic. Nevertheless, some empirical expressions that were basically derived in order to determine the interaction energy (or the enthalpy of formation) in the case of intermolecular hydrogen bonds are also used from time to time in the estimation of the energy of intramolecular hydrogen bonds. In the following subsections, I will discuss the two most common approaches that are based on spectroscopic quantities, namely the red-shift of the X-H proton-donor stretching vibration frequency and the proton downfield shift in the ^1^H NMR spectrum.

#### 2.6.1. Iogansen’s Relationship

Based on the results obtained for various phenol complexes, in 1969 Iogansen and Rassadin proposed an empirical formula for the relationship between intermolecular hydrogen bond energy (the hydrogen bond enthalpy of formation) and the red-shift of the X-H stretching vibration frequency ($\Delta \nu_{\mathrm{XH}}$) that takes place upon the hydrogen bond formation [150,151]

(36) $$E_{\mathrm{HB}}=0.33\sqrt{\Delta \nu_{\mathrm{XH}}-40}>0$$

The $\Delta \nu_{\mathrm{XH}}$ red-shift can be obtained either from experimental measurements or theoretical computations. Unfortunately, although the Iogansen’s relationship was derived for intermolecular hydrogen bonds [150,151], it is also used in order to estimate intramolecular hydrogen bond energies [119,152,153,154], where its applicability is at least unclear. One such use will be discussed in more detail here.

Using the formula (36), Nikolaienko et al. [119] estimated the energies of an impressively large number (However, it is highly doubtful that all of the more than 4000 conformers studied there actually correspond to true minima on the potential energy hypersurface and thus it is unclear what these conformers really mean.) of O-H⋯O, O-H⋯N, N-H⋯O, and O-H⋯C intramolecular hydrogen bonds in some biologically relevant DNA-related molecules. It is obvious that the use of this expression requires the knowledge of the reference vibration frequency $\nu_{\mathrm{XH}}^{\mathrm{free}}$. Despite the fact that, in the case of an isolated molecule, obtaining such a quantity is, at least in terms of theoretical calculations, a fairly simple process; however, in the case of intramolecular interaction, it is controversial which frequency should be best taken as a reference [40,41]. It is enough to mention here a very common problem with coupling vibrations. Anyway, Nikolaienko et al. [119] stated that “$\nu_{\mathrm{XH}}^{\mathrm{free}}$ has been calculated as the simple average of stretching vibration frequencies for XH groups, such that: (i) their H atom does not participate in any XH⋯Y bonding (i.e., no QTAIM bond path ends on it except for the one corresponding to the XH covalent bond), and (ii) unique normal vibration exists with $c_{j}^{\mathrm{XH}}>c_{\mathrm{th}}$.”, where $c_{j}^{\mathrm{XH}}=\partial l_{\mathrm{XH}}/\partial x_{j}$ ($x_{j}$ is the *j*-th normal coordinate) and $c_{\mathrm{th}}$ is the fixed treshold value (=0.92). Subsequently, based on the thus calculated energy values, these authors obtained, for each type of the hydrogen bond under consideration, a relationship with the determined value of the electron density at the bond critical point of a given hydrogen bond, $E_{\mathrm{HB}}=A\rho_{\mathrm{BCP}}+B$. The linear fit values for A and B thus obtained (the signs have been changed, so that the resulting $E_{\mathrm{HB}}$ values are negative) by Nikolaienko et al. [119] for each of the types of hydrogen bonds considered by them are shown in Table 5.

In principle, one could complain that the *B* values should be exactly zeros, as a zero electron density value should result in no hydrogen bonding and, therefore, also zero energy value. On the other hand, however, a significant portion of the electron density in the BCP is only due to the mutual overlapping of atomic orbitals of various atoms, and not only H and Y [107]. This fact was completely ignored here. Most likely, a correction is required to at least subtract the electron density contributions from H and Y, and perhaps even X. A somewhat similar correction has recently been proposed by Scheiner for calculating corrected NMR chemical shift for a proton involved in an intramolecular hydrogen bonding [141].

#### 2.6.2. Chemical Shift—Based Method

As mentioned earlier, one of the characteristic effects accompanying the formation of a hydrogen bond is the ^1^H NMR signal shift for the donor proton, i.e., the so-called downfield shift or proton magnetic deshilding, Δδ [138,139,140,141]. As early as in 1961, Gränacher [155] noticed a linear correlation between the proton chemical shift and the shift of the infrared absorption band, announcing the possibility of obtaining values of intermolecular hydrogen bond energies via the following equation [145]

(37) $$E_{\mathrm{HB}}=\Delta \delta +\left(0.4\pm 0.2\right)>0$$

Quite recently, this expression was used by Afonin et al. [121] in order to estimate energies of many different intramolecular hydrogen bonds, including improper, blue-shifting [156,157] ones, the energy of which cannot be estimated while using Equation (36). Importantly, their aim was to compare the energies that were obtained in this way with their counterparts obtained using other popular methods of estimating the energy of hydrogen bonds. Moreover, this approach allowed them to obtain new correcting parameters in the formulas combining the hydrogen bond energy with the local potential energy density ($V_{\mathrm{BCP}}$) and the electron density ($\rho_{\mathrm{BCP}}$) at the BCP of these interactions and determine the quality (However, the combination of experimental rather than theoretical ^1^H NMR data with theoretically determined QTAIM parameters for theoretically obtained geometries of the molecules under consideration is somewhat suspicious.) of the methods that are based on these parameters [121]. In this way, Afonin et al. showed that the estimates based on geometric parameters [158,159,160] are very poor (therefore, they are not discussed in this review). Nevertheless, even less reliable (2–3 times overestimation) values were obtained while using the uncorrected Espinosa’s formula, whereas applying a multiplier of 0.31 significantly improved the results. Based on the values of the hydrogen bond energies that were obtained by Equation (37) and the calculated values of either $V_{\mathrm{BCP}}$ or $\rho_{\mathrm{BCP}}$, Afonin et al. [121] obtained Equation (33) for the former parameter and $E_{\mathrm{HB}}=-191.4\;\rho_{\mathrm{BCP}}+1.78$ for the latter one, where the coefficients A and B are noticeably close to those that were obtained earlier by Nikolaienko et al. [119] (see last column in Table 5).

## 3. Summary

This article is a comprehensive critical overview of the currently most commonly used theoretical methods for estimating the energy of intramolecular hydrogen bonds and other intramolecular interactions. All of these methods have been grouped, as follows: conformational methods (The Open-Closed Method, Ortho-Para Method, Related Rotamers Method, Geometry-Corrected Method, Geometry-Corrected Related Rotamers Method), Rotation Barriers Method, Dimer Method, Isodesmic Reactions Method, QTAIM-based methods (Espinosa’s Method and IQA-based method) and empirically-based methods (Iogansen’s relationship and chemical shift - based method). The main emphasis is placed on two issues, namely the theoretical rationale of a given method and the fact that within the adopted method its diverse variants are often possible. Quite often, the methods themselves and their variants may lead to a wide range of the estimates being obtained by them.

The user should be aware that the applicability of a particular method is quite often very limited. This is especially seen in the case of conformational methods. Urgent attention should be paid that the reference form or forms do not have significant new stabilizing (attractive) or destabilizing (repulsive) interactions and that the structure of such forms is as close as possible to the intramolecularly hydrogen-bonded form. Because of the fact that ensuring such conditions is not always a simple task, the emerging methods of a non-invasive estimation of the intramolecular hydrogen bond interaction energy, i.e., not requiring any open form with the intramolecular hydrogen bond (or any other interaction) of interest being broken, are particularly important.

The performed energy estimates should be completed with an evaluation of their credibility. This can be done by either showing quite good correlations with various types of parameters used for indirect assessment of the bond strength or by an in-depth comparison of the obtained estimates for structurally similar systems. It is also recommended to use several estimation methods simultaneously. It should also be remembered that most of the problems that are encountered result from the fact that just trying to estimate the energy of intramolecular hydrogen bonding is also an attempt to introduce an indefinable quantity.

## Figures and Tables

**Table 1:** Determined (B3LYP/aug-cc-pVTZ) energy values (in kcal/mol) of Si-H⋯Al interactions in $1_{c}$, $2_{c}$ and $3_{c}$ (see Figure 6).

System	Rotated Group	SP	P1	P2	P3	P4	P5	OPT
**1**	−SiH_2_	−5.31	−4.88	−4.41	−3.36	−1.50	−0.99	−1.08
	−AlH_2_	−7.01	−6.61	−5.79	−4.41	−3.24	−1.75	
**2**	−SiH_2_	−5.23	−4.75	−3.93	−2.46	−2.42	−2.42	$p_{\pi }\to$ Al
	−AlH_2_	−8.09	−7.37	−6.27	−4.85	−4.73	−4.72	
**3**	−SiH_2_	−6.04	−5.03	−3.47	−1.41	−1.40	−1.40	−4.97/−0.75
	−AlH_2_	−10.61	−10.05	−8.44	−6.23	−6.19	−6.19	

**Table 2:** Some energetic (in kcal/mol) and geometric (in Å) parameters computed (MP2/6-311++G**) for different forms of 3-aminoacrolein (Y = O) and 3-aminopropential (Y = S).

Y	$E^{\mathrm{EZ}}$	$E^{\mathrm{EE}}$	$E^{\mathrm{ZE}}$	$E_{\mathrm{HB}}^{\mathrm{OCM}}$	$E_{\mathrm{HB}}^{\mathrm{GCM}}$	$d_{H\cdots H}^{\mathrm{ZE},f}$	$d_{H\cdots H}^{\mathrm{ZE}}$	$\Delta d_{H\cdots H}^{\mathrm{ZE},f\to \mathrm{ZE}}$	${\tilde{\tilde{\Delta }}}_{\mathrm{rel}}$
O	4.77	3.77	6.50	−6.50	−5.28	1.840	2.141	−0.301	−1.87
S	6.07	4.09	6.02	−6.02	−6.96	1.968	2.127	−0.159	−1.86

**Table 3:** Estimated values (MP2/6-31G**) of intramolecular O-H⋯O hydrogen bond energies (in kcal/mol) in malondialdehyde and acetylacetone [33].

Molecule	$E_{\mathrm{HB}}^{\mathrm{OCM}-D}$	$E_{\mathrm{HB}}^{\mathrm{OCM}-A}$	$E_{\mathrm{HB}}^{\mathrm{RBM}-D1}$	$E_{\mathrm{HB}}^{\mathrm{RBM}-D2}$	$E_{\mathrm{HB}}^{\mathrm{RBM}-A1}$	$E_{\mathrm{HB}}^{\mathrm{RBM}-A2}$
malondialdehyde	−14.0	−10.7	−14.1	−14.0	−12.4	−12.9
acetylacetone	−16.2	−13.3	−15.1	−16.9	−12.3	−14.5

**Table 4:** Interaction energies (kcal/mol) of the X⋯O intramolecular contact in ZZ-3-halogenopropenal estimated by means of several dimers utilized in Dimer Model (DM) [74].

X	$E_{X\cdots O}^{\mathrm{ZE}\cdots \mathrm{EZ}}$	$E_{X\cdots O}^{\mathrm{ZE}\cdots \mathrm{EE}}$	$E_{X\cdots O}^{\mathrm{XMe}\cdots \mathrm{Fa}}$	$E_{X\cdots O}^{\mathrm{XAc}\cdots \mathrm{Fa}}$
F	1.82	1.97	0.52	0.44
Cl	2.98	3.34	1.06	0.38
Br	3.49	3.95	1.08	0.19

**Table 5:** Linear fit parameters for the linear relation $E_{\mathrm{HB}}=A\rho_{\mathrm{BCP}}+B$ between the hydrogen bond energy (in kcal/mol) and electron density at the BCP (au) of the indicated hydrogen bond [119].

Type	A	B	R	$R^{2}$
O-H⋯O	−239 ± 2.2	3.09 ± 0.07	0.93	0.86
O-H⋯N	−142 ± 2.1	−1.72 ± 0.08	0.97	0.94
N-H⋯O	−225 ± 12	2.03 ± 0.25	0.85	0.72
O-H⋯C	−288 ± 19	0.29 ± 0.22	0.86	0.74
together	−200 ± 2.2	1.70 ± 0.07	0.88	0.77

## Abbreviations

The following abbreviations are used in this manuscript:

OCM	Open-Closed Method
opM	*Ortho*-*Para* Method
RRM	Related Rotamers Method
GCM	Geometry-Corrected Method
GCRRM	Geometry-Corrected Related Rotamers Method
RBM	Rotation Barriers Method
DM	Dimer Model
IRM	Isodesmic Reactions Method
EM	Espinosa’s Method
QTAIM	Quantum Theory of Atoms in Molecules
IQA	Interacting Quantum Atoms
BP	bond path
BCP	bond critical point
